# Assessing the Contribution of Marine Protected Areas to the Trophic Functioning of Ecosystems: A Model for the Banc d’Arguin and the Mauritanian Shelf

**DOI:** 10.1371/journal.pone.0094742

**Published:** 2014-04-11

**Authors:** Sylvie Guénette, Beyah Meissa, Didier Gascuel

**Affiliations:** 1 UMR Agrocampus Ouest, Inra, Ecologie et santé des écosystèmes, Université Européenne de Bretagne, Rennes, France; 2 Institut Mauritanien de Recherches Océanographiques et des Pêches (IMROP), Nouadhibou, Mauritanie; Aristotle University of Thessaloniki, Greece

## Abstract

Most modelling studies addressed the effectiveness of marine protected areas (MPA) for fisheries sustainability through single species approach. Only a few models analysed the potential benefits of MPAs at the ecosystem level, estimating the potential export of fish biomass from the reserve or analysing the trophic relationships between organisms inside and outside the MPA. Here, we propose to use food web models to assess the contribution of a MPA to the trophic functioning of a larger ecosystem. This approach is applied to the Banc d’Arguin National Park, a large MPA located on the Mauritanian shelf. The ecosystem was modeled using Ecopath with Ecosim, a model that accounts for fisheries, food web structure, and some aspects of the spatial distribution of species, for the period 1991–2006. Gaps in knowledge and uncertainty were taken into account by building three different models. Results showed that the Banc d’Arguin contributes about 9 to 13% to the total consumption, is supporting about 23% of the total production and 18% of the total catch of the Mauritanian shelf ecosystem, and up to 50% for coastal fish. Of the 29 exploited groups, 15 depend on the Banc for more than 30% of their direct or indirect consumptions. Between 1991 and 2006, the fishing pressure increased leading to a decrease in biomass and the catch of high trophic levels, confirming their overall overexploitation. Ecosim simulations showed that adding a new fleet in the Banc d’Arguin would have large impacts on the species with a high reliance on the Banc for food, resulting in a 23% decrease in the current outside MPA catches. We conclude on the usefulness of food web models to assess MPAs contribution to larger ecosystem functioning.

## Introduction

Marine protected areas (MPA) are often viewed as an effective tool to protect aquatic resources from overexploitation and other anthropic sources of degradation [1]–[4]. Several reviews of field studies on marine reserves worldwide showed benefits for a wide array of species, especially for large predators [5]–[8]. In addition, due to fish movements, biomass accumulated inside MPAs may be exported ouside. Such a spillover effect [9], [10] were demonstrated in many places and may lead to an increase in catch, especially along the MPA borders [11]–[13]. Nevertheless, a few field studies found that spillover effects were highly localised [14], [15]. Several modelling studies showed that migrations and/or nomadic movements could lower or even negate the conservation effects of MPAs and that fish stocks may only benefit from protected areas of sufficient size, e.g. [14], [16]–[18]. Some ecosystem models suggest that biomass exports would be of the same order of magnitude as the amount of catch that could be obtained inside the reserve [19].

Most modelling studies addressed the effectiveness of closed areas for fisheries sustainability through single species approaches and taking into account three major mechanisms: the spillover effect, the potential export of larvae, and the reduction in the overall fishing mortality which may occur when MPA are located on strong fish aggregations [20], [21]. Only a few models addressed the potential benefits of MPAs at the ecosystem level, estimating the potential export of fish biomass from the reserve [19] or analysing the trophic functioning (the trophic relationship between functional groups) inside the MPA [22]–[26]. In both cases, the models do not consider the effects fishing restrictions may have on the entire food web in the area around the MPA. Also, only a few models addressed the potential regional benefit of MPAs by modelling the region instead of the MPA alone [24], [27]. In the study area, two ecosystem models have been built for the entire EEZ [28] or the Banc d’Arguin by itself [29], but never with the intention of testing the role of the Banc d’Arguin as an MPA.

We propose to use food web models to assess the contribution of MPAs to the trophic functioning of a larger ecosystem using the Banc d’Arguin National Park (Mauritania), a large MPA covering 20% of the whole Mauritanian shelf, as a case study. This shelf is enriched by an upwelling and is considered as one of the most productive area worldwide, with catches of about one million tonnes per year. The Banc d’Arguin is suspected to constitute a major nursery for several species [30] and to sustain a large part of the Mauritanian marine production.

We used the widely known Ecopath with Ecosim modelling tool EwE version 6.3 [31] to build a model that includes trophodynamics, fisheries and some aspects of spatial structures linked to habitat. We modelled the period 1991-2006 a period for which catches were compiled and data from regular scientific surveys were available. We used scenarios of fishing and habitat loss inside the Banc to gain insight in its importance in the total production of the ecosystem and its links with the rest of the shelf.

## Methods

### The study area

**Figure 1:**
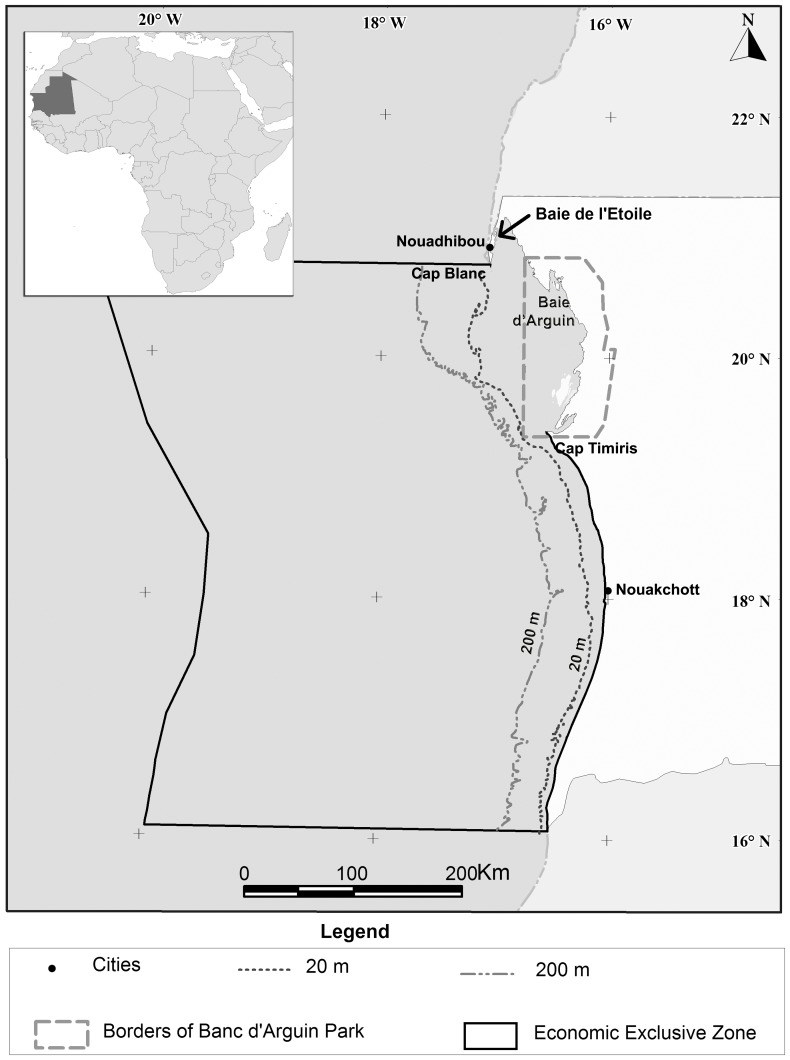
The Mauritanian shelf (the study area) and the Banc d’Arguin National Park (delimited with the black line). The study area covers 33,224^2^ including the Banc d’Arguin (6,450 km^2^).

The Mauritanian EEZ is located in the Northeast Atlantic from 16°04’N to 20°46’N and the study area (<200 m) covers about 33,224 km^2^ including the Banc d’Arguin [32] (Figure 1). The shelf is enriched by an upwelling that is permanent around Cap Blanc but spanning only 9 months around Nouakchott. The Banc d’Arguin National Park (hereby called the Banc) is a marine protected area of 6,450 km^2^, the largest in Africa, which encompasses large beaches, tidal flats and seagrass beds. The National Park was created in 1976 to protect the habitat for migratory birds, but its objectives have been broadened since to protect the production of its habitats and contribute to the sustainable development of resource exploitation and economic development of the Park residents.

Mauritania has no long-standing fishing tradition except for the artisanal fishing of mullets (*Mugil cephalus*, *Mugil capurrii*) in the Banc during their migration in the area [33]. The Banc is partially closed to fishing, allowing only the Park residents (the Imraguens) to fish with small sailboats and accounting for about 5% (4,000 t) of the artisanal fishery in 2006. The domestic small-scale (artisanal) fishery developed during the 1990s [34] exploits mainly mullets, coastal selacians (e.g. *Rhinobatus rhinobatos*, *Mustellus mustellus*), meagre (*Argyrosomus regius*), octopus (*Octopus vulgaris*), catfish (*Arius heudelotii*) and various other demersal (e.g. Sparidae, Serranidae, Pomadasyidae, Pleuronectiformes), and pelagic (e.g. *Scomberomorus tritor, Caranx rhonchus*) fish.

Catches from the EEZ are mainly from foreign vessels from the European Union (Spain, France, Greece, Italy, Netherlands), Russia, and several others, targeting both demersal and pelagic species (Institut Mauritanien des Recherches Océanographiques et des Pêches, IMROP) [35]. The pelagic fleet fish mainly on horse mackerel (*Trachurus trecae, T. trachurus*) and clupeids (*Engraulis encrasicolus, Sardina pilchardus, Sardinella aurita, S. maderensis*). The demersal fishery exploits cephalopods (squid, cuttlefish and octopus), shrimps, hake (*Merluccius merluccius, M. polli, M. senegalensis*), and various other demersal fish from the shelf and the continental slope. The rapid increase in effort observed in the 1970s resulted in a corresponding increase in landings from around 500,000 t in 1970 to almost one million t in 2000. This increase in exploitation resulted in a documented 75% decline in demersal resources since 1982 [32], [36].

### Model structure

Ecopath models include the following information on each functional groups: biomass, production and consumption per unit of biomass (P/B and Q/B respectively) (see Table 1 and Text S1 in File S1), and diet composition (Table S2 in File S1). Ecosim, the temporal dynamic portion of the software describes changes in biomass and flows as environmental conditions and fishing are modified. The base model was constructed for the year 1991 and the time series span the period 1991–2006. The Ecosim model was driven by fishing effort for each fleet: artisanal, industrial demersal and pelagic (Table S3 in File S1) and fit to the observed biomasses and landings by minimizing the sum of squares of differences between model predictions and observations of biomass and catches (see Text S1 and Table S7 in File S1).

**Table 1:** Input and output parameters for the Base Ecopath model.*

										Catch (t/km^2^)			
	Group name	TL	Biom (t/km^2^)	Z (/year	P/B/year	Q/B/year	EE	P/Q	BA	Artis.	Dem.	Pel.	Total
Migratory													
1	Marine mammals	**4.16**	0.01		0.04	12.5	**0.000**	**0.003**	0	0	0	0	0
2	Coastal birds	**3.44**	0.01		0.28	67.0	**0.000**	**0.004**	0	0	0	0	0
	**Meagre**												
3	Meagre ad	**3.94**	0.12	0.21	0	2.1	**0.657**	**0.100**	0	0.007	0.007	0	0.014
4	Meagre juv	**3.80**	**5.E-05**	0.3	0	**17.9**	**0.974**	**0.017**	0	0	0	0	0
5	Mullets	**2.10**	**0.42**		0.8	8.2	0.800	**0.097**	0	0.04	0.063	0	0.103
Pelagics													
6	Pelagic L	**3.84**	**3.42**		0.96	5.4	0.900	**0.178**	0	0.008	0	2.671	2.679
7	Mackerel	**3.20**	1.45		0.45	3.0	**0.735**	**0.150**	0	0	0	0.289	0.289
8	Sardine	**2.90**	11.79		0.65	4.3	**0.771**	**0.150**	0	0	0	1.801	1.801
9	Sardinelles	**2.78**	18		0.99	7.7	**0.785**	**0.129**	0	0.108	0	2.202	2.31
10	Horse mackerels	**3.23**	10		0.72	**3.6**	**0.844**	0.200	0	0.003	0	3.809	3.812
Coastal													
11	Coastal selacians	**3.64**	1.24		0.3	**2.0**	**0.015**	0.150	-0.05	0.046	0.017	0	0.063
12	Coastal M	**3.05**	0.83		0.58	**2.9**	**0.858**	0.200	0	0.053	0.068	0	0.121
13	Coastal S	**3.12**	**4.21**		0.62	**3.1**	0.950	0.200	0	0	0	0	0
	**Croakers**												
14	Croakers ad	**3.66**	0.077	0.6		3.9	**0.754**	**0.156**	0	0.001	0.002	0	0.003
15	Croakers juv	**3.23**	*0.004*	1.17		*9.9*	**0.706**	**0.118**	0	0	0	0	0
	**Seabreams**								0				
16	Seabreams ad	**3.15**	1.69	0.48		4.7	**0.884**	**0.102**	0	0.044	0.123	0	0.167
17	Seabreams juv.	**3.14**	*0.012*	0.76		*21.1*	**0.884**	**0.036**	0	0	0	0	0
	**Catfish**												
18	Catfish ad	**3.48**	0.6	0.34		4.1	**0.226**	**0.083**	0	0.034	0	0	0.034
19	Catfish juv	**3.05**	0.002	0.58		22.3	**0.780**	**0.026**	0	0	0	0	0
Shelf													
20	Shelf selacians	**4.06**	0.2		0.24	**1.6**	**0.787**	0.150	0	0.001	0.01	0	0.011
21	Shelf L	**4.08**	0.36		0.47	3.4	**0.467**	**0.140**	0	0	0.071	0	0.071
22	Shelf M	**3.24**	1.55		0.57	6.2	**0.908**	**0.092**	0	0.01	0.136	0	0.146
	**Groupers**												
23	Groupers ad	**3.78**	0.11	0.43		3.2	**0.938**	**0.137**	-0.05	0.017	0.008	0	0.025
24	Grouper juv	**3.53**	0.0004	0.44		16.2	**0.536**	**0.027**	-0.05	0	0	0	0
	**Sparids**												
25	Sparids ad	**2.96**	1.29	0.44		2.4	**0.871**	**0.186**	0	0.009	0.005	0	0.014
26	Sparids juv	**2.60**	0.01	0.86		9.8	**0.939**	**0.088**	0	0	0	0	0
27	Scianids	**3.42**	0.22		0.29	4.3	**0.665**	**0.068**	-0.05	0.001	0.016	0	0.017
28	Shelf soles	**3.31**	0.35		0.58	**2.9**	**0.882**	0.200	0	0.001	0.008	0	0.009
29	Shelf S	**3.05**	**6.195**		0.82	7.6	0.950	**0.108**	0	0	0.005	0	0.005
30	Octopus vulgaris	**3.15**	1.37		1.4	**4.7**	**0.632**	0.300	-0.03	0.218	0.665	0	0.883
31	Cephalopods	**3.56**	1		1.2	**4.0**	**0.839**	0.300	-0.03	0.001	0.254	0	0.255
Sedentary													
32	BA L crustaceans	**2.43**	9.12		1.44	**7.2**	**0.877**	0.200	0	0	0	0	0
33	BA molluscs	**2.05**	17.86		1.5	**16.7**	**0.888**	0.090	0	0	0	0	0
34	BA worms	**2.03**	5.32		3	**33.3**	**0.805**	0.090	0	0	0	0	0
35	BA crustaceans	**2.10**	1.14		2.4	**12.0**	**0.975**	0.200	0	0	0	0	0
36	BA other inverts	**2.21**	0.57		1.8	**9.0**	**0.868**	0.200	0	0	0	0	0
37	BA meiobenthos	**2.00**	2.09		9	**100.0**	**0.930**	0.090	0	0	0	0	0
38	shelf L crustaceans	**2.52**	8.1		1.5	7.5	**0.732**	**0.200**	0	0.001	0.004	0	0.005
39	shelf molluscs	**2.00**	26.21		1.5	**16.7**	**0.500**	0.090	0	0	3E-06	0	3E-06
40	shelf worms	**2.00**	31.77		3	33.0	**0.409**	**0.091**	0	0	0	0	0
41	shelf crustaceans	**2.10**	8.04		2.4	12.0	**0.797**	**0.200**	0	0	0	0	0
42	shelf other inverts	**2.10**	17.21		1.8	9.0	**0.224**	**0.200**	0	0	0	0	0
43	shelf meiobenthos	**2.00**	8.91		9	**100.0**	**0.253**	0.090	0	0	0	0	0
44	mesozoopl.	**2.00**	55.08		24	112.0	**0.146**	**0.214**	0	0	0	0	0
45	macrozoopl.	**2.40**	3.41		4.3	17.0	**0.728**	**0.253**	0	0	0	0	0
46	BA mesozoopl.	**2.00**	**1.78**		24	112.0	0.800	**0.214**	0	0	0	0	0
47	BA macrozoopl.	**2.40**	**2.5**		4.3	17.0	0.800	**0.253**	0	0	0	0	0
48	BA phytopl.	**1**	5.9		100	0	**0.260**	0.000	0	0	0	0	0
49	phytoplankton	**1**	67.8		100	0	**0.853**	0.000	0	0	0	0	0
50	algae and eelgrass	**1**	548.8		4.06	0	**0.011**	0.000	0	0	0	0	0
51	Detritus	**1**	560		0	0	**0.455**	0.000		0	0	0	0

* Values estimated in Ecopath are in bold.TL: trophic level; Biom: biomass, Z: total mortality; P/B and Q/B: production and consumption per unit of biomass; EE: ecotrophic efficiency; P/Q: gross efficiency; BA: biomass accumulation; Artis. Dem. Pel. are the artisanal, demersal and pelagic fleets.

The 1991 base model (Table 1) is structured around habitat preferences and degree of mobility which guide what functional groups can feed upon. Based on the sedentarity of most zoobenthos and the knowledge that phytoplankton species composition differs inside and outside the Banc [37], the primary and secondary producers were separated into two habitats: the shelf and the Banc d’Arguin (BA). The name of these groups always starts with habitat preference. The allocation of these prey functional groups in the diet of higher trophic-level groups (fish, cephalopods, mammals and birds) was based on the spatial distribution of predators. The value *pBA_i_^base^* is the proportion of benthic and pelagic invertebrates (*i*) from the Banc that are consumed by a functional group of predator in the base model (Table S8 in File S1).

The marine mammal group includes only species mostly feeding in the study area, ignoring oceanic species that feed marginally in the area [38] (Table S4 in File S1). Coastal birds are either breeders or migratory species spending part of the year in Mauritania, feeding in coastal waters or on muddy flats in the Banc d’Arguin (Table S5 in File S1).

Fish were classified by habitat preferences: coastal, shelf, pelagic, and migratory (Table 1; see Table S1 for species composition in File S1). Coastal species are found in the Banc d’Arguin as well as on the near shelf and along the coast south of the Banc. Shelf species prefer deeper waters more offshore and including the break of the shelf. Migratory fish species (meagre and mullets) are coastal species that migrate along the coast of Mauritania and Senegal. Pelagic species also carry out seasonal migrations between Morocco and Senegal and can be found on the whole shelf, including nearshore waters. Sardinelles are the most coastal of pelagic species, captured in the Banc for part of the year although the nursery is located on the coast south of the Banc [39]. Horse mackerels are classified as pelagics here because they are surveyed with other pelagic species although they are benthopelagics, feeding on both demersal and pelagic species, and partially in the Banc. Fish species have been further grouped according to their size (small (S), medium (M) and large (L)), production, diet and commercial importance. Six groups of coastal fish (meagre, croakers, seabreams, catfish, groupers, and sparids), were divided into two phases: juveniles (age 0–1 year) and adults. Except for groups designating fish of a particular family or one species, fish groups names are typically composed of first, their habitat preference, and second, their size. The sampling of juvenile fish in the Banc [30] seem to support the role of the Banc as a nursery but is not sufficient to assess the importance for fish population as there are other habitats available outside the Banc (e.g., Baie du Lévrier, [40], [41]). Fish biomasses were calculated from acoustic and trawl survey (Text S1 in File S1), catches were obtained from IMROP database for each fishing fleet (artisanal, industrial pelagic and demersal) which contains no information on discards.

The biomasses of shelf macrobenthos were obtained from two transects (north and south) estimates crossing the shelf from the Banc to the limit of the shelf [42], using taxa specific conversion ratios from AFDW to WW [43] (see Table S6 in File S1). Polychaetes and molluscs were the main group found throughout the shelf. In the Banc d’Arguin, the biomass of benthic invertebrates was estimated only for tidal flats [44]. Thus, biomass from the intertidal area were empirically determined, according to various scenarios (see below). As a first approximation, meiobenthos biomass was assumed to be 6% of the macrobenthos biomass, the observed proportion for the shelf, but their biomass was increased to balance the model. P/B values were obtained from the literature [45]–[47] and P/Q values ( = gross efficiency) from [45]. Further details on the model construction, parameters and inputs are provided in the Supplementary material (Text S1 in File S1)

### Uncertainty and scenarios

In spite of the suspected ecological importance of the Banc d’Arguin, there is no estimate of biomass of benthic invertebrates in the subtidal area, a likely important source of food for juvenile and adult fish species. Thus, a sensitivity analysis was performed by building three models to represent different initial states of benthos biomass in the Banc. The base model (Table S8 in File S1) is considered the reference and assumes that the density (in weight) of subtidal benthos is equal to that of the intertidal invertebrates.

The second model (P30) was built assuming that the subtidal benthos density was 30% higher than that of the intertidal which allows for more fish to feed in the Banc. This is a conservative assumption given that the ratio of densities (in numbers) subtidal/intertidal in the Baie de l’Étoile benthos [41] amounts roughly to 1.8 (a ratio that would likely be too high to be applied directly to biomass). In this model, the proportion of feeding on benthos in the Banc (pBA_i_^P30^ for the group i) was increased from 50 to 100% for all juvenile groups, and by 50% for 7 other coastal functional groups (Table S8 in File S1). This change in pBA_i_ also accounts for the uncertainty regarding the importance of the Banc as a nursery for juveniles and coastal S fish. In addition, the biomass of coastal S, shelf S, and pelagic L were left to increase by replacing the high EE (0.9 for pelagic L and 0.95 for the others) required to balance the base model with a value of 0.8.

The third model (M30) supposed that the subtidal benthos is 30% less abundant and thus, reduces the possibility of feeding in the Banc. The proportion of benthos consumed in the Banc (pBA_i_^M30^) was reduced from 50% in the base model to 25% in M30, with the same fish biomasses. Each Ecosim model was fitted to the 1991–2006 time series separately (See Text S1 in File S1 for detail of the balancing and fitting process).

Starting from 1991 in each of these fitted models, scenarios of exploitation were ran forward over a period of 50 years to assess the theoretical long-term impact of: 1. a hypothetical loss of habitats in the Banc; 2. a hypothetical new fishing fleet (Fictive fleet) operating in the Banc. The habitat loss was obtained by applying an additional initial mortality (M = 0.02) on groups located inside the Banc (benthic invertebrates, plankton and seagrass; i.e. groups 32–37, 47–49, 51) that increased rapidly causing a ∼40% decline in seagrass biomass and the extinction of the other nine groups.

The simulations for the fictive fleet are carried out by adding a new fishing mortality for each exploited groups. In order to mimic a fleet targeting mainly the species feeding inside the Banc d’Arguin, these mortalities are assumed to be in the same proportion as that of the invertebrates consumed in the Banc:



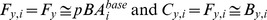
(1) where *F_y,i_*, *C_y,i_* and *B_y,i_* are the additional fishing mortality, the catch of the fictive fleet and the biomass, for group *i* in year y.

Simulations are initialized using a low value for F_y_ (F_1991_ = 0.02) to ensure minimal disturbance to the balanced model. Then, simulations use an effort time series that doubles every year until F_y_ reaches a maximum of 0.4 for juveniles, coastal S and shelf S, less targeted by fisheries due to their small body sizes, and *F_y_* = 0.8 for the others. Since the proportion of invertebrates from the Banc in fish diets differs for each model (*pBA_i_^base^*, pBA_i_^M30^, and *pBA_i_^P30^*), the simulated *F_y,i_* and *C_y,i_* also vary with each model.

Simulations were performed 50 years longer than the 16 years covered by the time series (1991–2006), and compared to the projection of current fishing mortalities (Status quo, Sq) in 2056. The ecosystem is assumed to have reached equilibrium state for the last simulated year. The effects of the fictive fleet were assessed using the ratio of the end biomass with and without the fictive fleets (B_fict_/B_Sq_), and the effects of habitat loss in the Banc with the ratio of the end biomass with the loss over the status quo biomass (B_Loss_/B_Sq_).

### Ecosystem structure and contribution from the Banc

Direct and indirect interactions within the ecosystem were analyzed using the Mixed Trophic Impact (MTI) routine of Ecopath, which assesses the relative impact of a slight increase in abundance of any group on the biomass of other groups in the food web [31]. The MTI index, scaled from -1 to 1, was calculated for every functional group (see Text S1 in File S1 for detail). Using the groups with absolute values larger than 0.1 resulted in a simplified food web featuring the most impacting functional groups. The results were used to show the links between habitats.

The contribution from the Banc was estimated using the parameters from the balanced model (consumption, production, mortality, etc.; see Table 1). The dependency of each functional group on the Banc d’Arguin was assessed by calculating the percentage of the consumption and the production originating from the Banc, from direct consumption on benthic and pelagic invertebrates and phytoplankton (groups 32–37, 46–48, the only true sedentary groups) and from indirect foodweb pathways.

The direct consumption in the Banc for predator *j* is calculated as: 



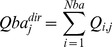
(2)where Q*_j,i_* is the consumption of prey *i* by predator *j*, *Nba* the number of invertebrate groups resident in the Banc. The contribution of the Banc’s invertebrates to the whole food web also comes from the consumption through indirect pathways involving other predators, the indirect consumption. It is calculated in successive steps.

1. The production of predator *j* based on direct consumption in the Banc that can thus be considered as originating itself from the Banc is:
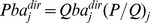
(3)where P/Q is the ratio of production per unit of consumption per year (Table 1).
2. The fraction of this production, issued from predator *j* and consumed by secondary predators (i.e. the part of the production originating from the Banc not going to detritus (*EE_i_*), not fished (*1-F_i_*) and not accumulated (1-*Bacc_i_*)) is:
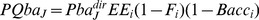
(4)
3. Thus, the indirect consumption (i.e. the consumption of secondary predator *j* on preys *i* which were considered as predators in the previous steps), originating from the Banc is:
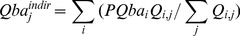
(5)where *PQba_i_* is the consumption on prey *i* originating from the Banc, while the *Q/∑Q* ratio expresses the part of predator *j* in all the consumption made on prey *i*.
4. Lastly, the total consumption originating from the Banc for a given predator is:
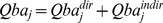
(6)

Equations 3 to 6 allow taking into account the “second level” predation, i.e. predators eating prey that feed in the Banc. Higher levels of predation may also exist, i.e. predators eating prey that eat prey feeding in the Banc, etc. This is computed replacing *Qba_j_^dir^* by *Qba_j_* in equation 5 and repeating equations 3 to 6 iteratively until stabilization.

The production of a given Ecopath group is proportional to its consumption, while the catch can be considered as the harvest of a fraction of the production. Therefore, the proportions of consumption, production and catch originating from the Banc for the group *j* (*pQba*_j_, *pPba_j_* and *pYba_j_* respectively) can be estimated as:



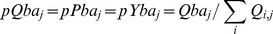
(7)


Finally, the proportions of the total consumption, production and catch originating from the Banc through direct and indirect paths are computed respectively with: 
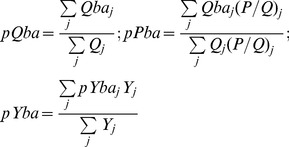
(8)


#### Trophic spectra

Results of the model are also presented using trophic spectra [48] based on the biomass and the mean trophic level from Ecopath. The biomass trophic spectrum is the graphical representation of the current distribution of the whole ecosystem biomass across trophic levels. It is built spreading the biomass of each Ecopath group across trophic levels around the group's mean trophic level, in order to represent the within group variability. According to the empirical method proposed in [49], [50], each group has its own distribution of biomass across trophic levels, defined by a log-normal function centered on each species' mean trophic level and using a standard deviation which is a measure of the within group variability of trophic levels, theoretically and conventionally defined. The trophic spectrum corresponds to the sum of all Ecopath groups, resulting is a single curve where species cannot be differentiated anymore, thus giving a simplified view of the ecosystem. We use the same approach to build a production trophic spectrum (from the Ecopath P/B ratios) and a catch trophic spectrum from which a fishing mortality trophic spectrum (F = C/B) was obtained.

## Results

### Food web characteristics

The simplified structure of the base model, focusing only on the species that have the largest impact on other species (|MTI| >0.1), indicates that the pelagic and demersal (shelf and coastal) compartments of the ecosystem overlap (Figure S1 in File S1). The pelagic food web is impacted mainly by pelagic L (large pelagics) and horse mackerels (hmack in Figure S1 in File S1) competing for sardines and sardinelles. In addition, horse mackerels also impact shelf S and both groups of macrozooplankton, while large pelagics also impact adult sparids and shelf S, very abundant demersal groups. The large pelagics group induces a trophic cascade by preying on zooplanktivorous fishes, which feed mainly on mesozooplankton, hence favouring large biomass of phytoplankton. Sardinelles compete with other pelagic species and thus have a negative impact on horse mackerel, sardine, and mackerel.

The demersal food web is impacted mainly by cephalopods, coastal selacians, and shelf L. Selacians, being an aggregate of numerous species with varied diets, feed ubiquitously on a large array of groups. Cephalopods benefit shelf selacians, an important predator, and impact negatively octopus and sparids adults through predation mortality. Abundant small demersals (shelf S, coastal S, sparids), are often in competition with other invertebrate feeders, fish (especially juveniles) or cephalopods. They also benefit several predators, namely groupers, croakers, shelf L, and shelf selacians. Since coastal birds are feeding mainly in coastal areas they impact several juvenile groups, including those of meagre, catfish, croakers, and seabreams.

### Dependency to the Banc d’Arguin

**Figure 2:**
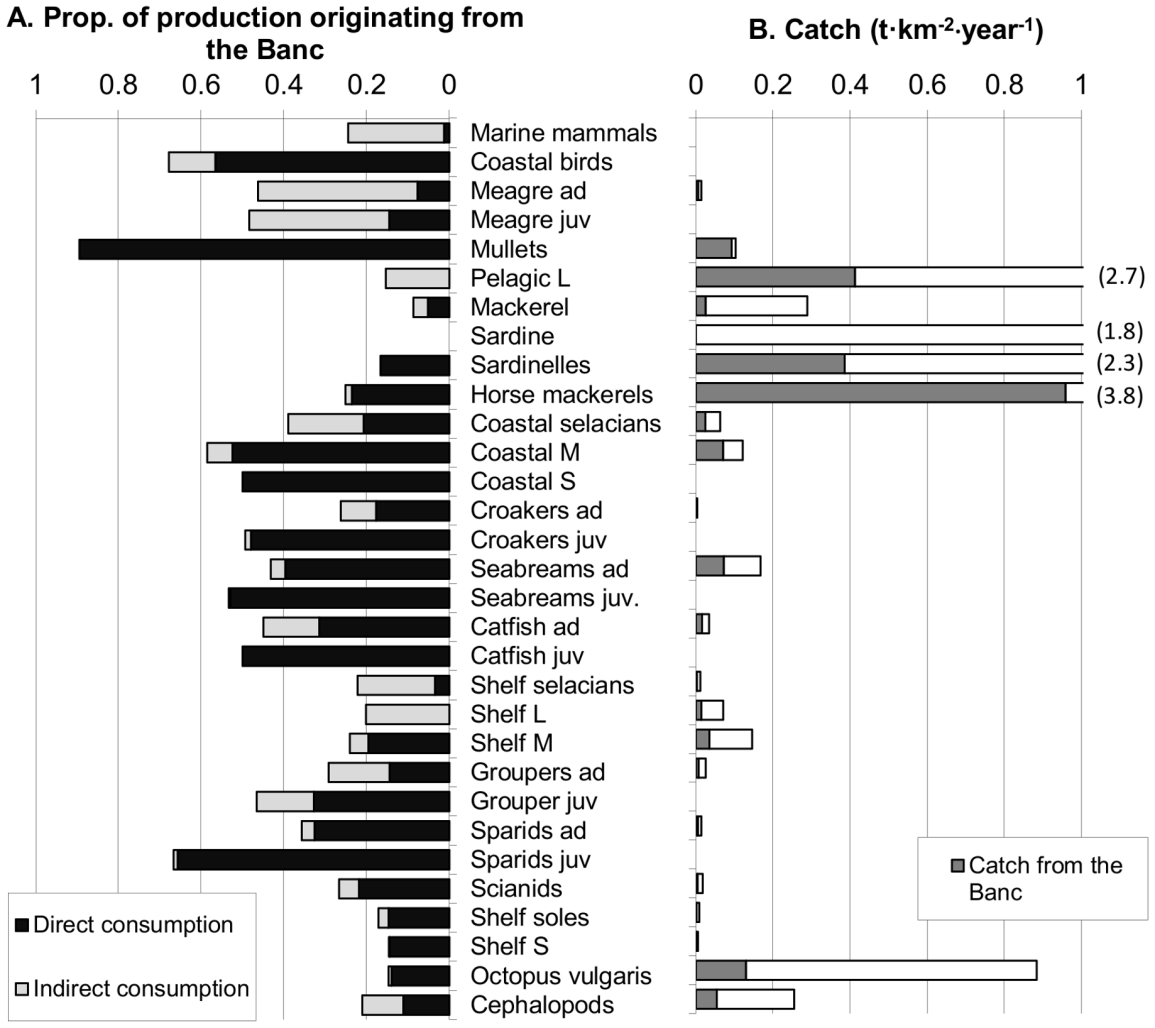
Portion of the production (A), and catches (t·km^−2^·year^−1^, (B) originating from the Banc d’Arguin, through direct or indirect pathways, for the 31 Ecopath groups of higher TL.

Almost all of the 31 high trophic level groups (gr 1 to 31: mammals, birds, fish and cephalopods) depend on the Banc d’Arguin for their consumption and thus for their production (Figure 2a). Sardine is the only exception because this group is assumed to eat exclusively phytoplankton and zooplankton outside the Banc. Fifteen groups, including all juvenile fish, depend on the Banc for more than 30% of their total production (and more than 50% for 8 groups), thus highlighting the role of the Banc as feeding and/or nursery grounds for many exploited species. The highest dependencies are observed for mullet (62%) and the coastal seabirds (66%). In general, dependency on the Banc is mainly due to direct consumption of invertebrates living in the Banc, especially in the case of coastal groups, including juveniles. Conversely indirect consumptions originating from the Banc through more or less complex pathways are important for marine mammals, meagre, large pelagics and selacians.

On average 10.5% of the total ecosystem consumptions are directly or indirectly originating from the Banc (Table 2). This estimate appears rather robust to the assumptions made in the modelling process, ranging from 9.4 to 12.6% for the alternative M30 and P30 models respectively. The consumption originating from the Banc is the basis of 7.9% of the total production for all animal groups together, and of 23.2% of the total production including primary producers (this high value for the latter estimate being due to the large amount of algae and eelgrass production in the Banc). For the 12 coastal fish groups (including mullets and meagre), the overall consumption and production from the Banc is higher than 50% (Figure 2a).

**Table 2:** Percent consumption (Q), production (P1 for animals only, P2 including primary producers) and catch (Y) of the Mauritanian shelf coming from invertebrates from the Banc d’Arguin (BA), both directly and indirectly through the food web (total): on the left for the Base, M30 and P30 models, and on the right for the various types of functional group (number of groups).

	All 47 groups together			Base model only		
	M30	Base	P30	Coastal (12)	Pelagics (5)	Shelf(12)
% Q from BA	9.4%	10.5%	12.6%	51.7%	14.2%	17.5%
% P1 fromBA	7.2%	7.9%	9.3%	51.1%	14.6%	17.7%
% P2 from BA.	23.1%	23.2%	23.4%			
% Y from BA	17.3%	18.1%	19.1%	55.9%	16.3%	17.7%

Taking into account the direct and indirect consumption of invertebrates from the Banc, 18.1% of the total yearly catch is estimated to originate from the Banc, (17.3 and 19.1% for the M30 and P30 models respectively) (Table 2). The largest catches originating from the Banc’s production are due to large pelagics, sardinelle and horse mackerel (Figure 2b) in spite of their low dependency on the Banc. This is because these three groups constitute a large biomass and a large proportion of the total catch. Similarly, 14.7% of the octopus catch, another very important species for Mauritanian fisheries, comes from the Banc's production. Demersal finfish catches are smaller in volume but their dependency on the Banc productivity reaches 44% on average, with mean values close to 24% for fishes distributed on the shelf and 56% for coastal fishes.

**Figure 3:**
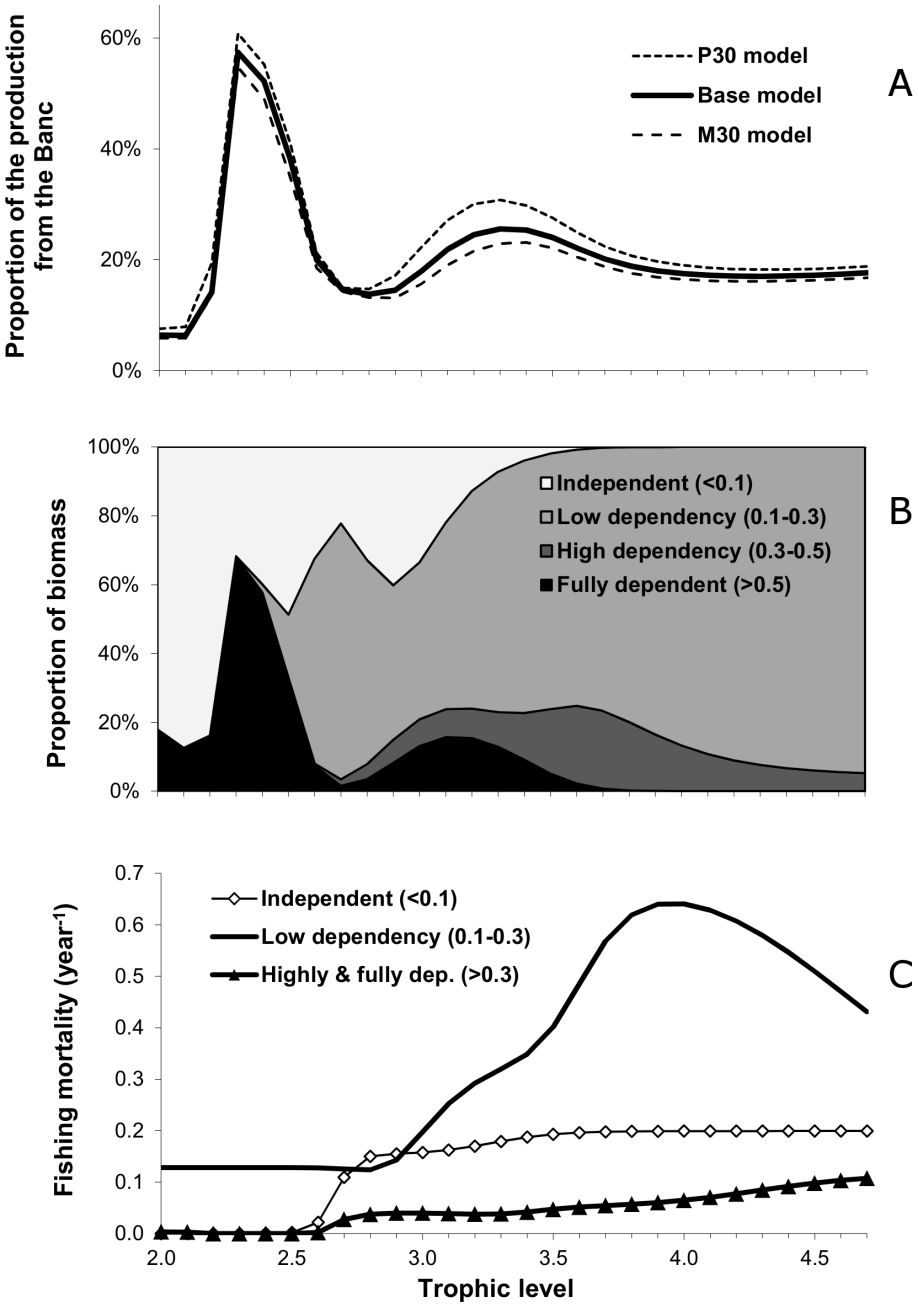
Trophic spectra on the dependency of the Mauritanian EEZ ecosystem to the Banc d’Arguin: A. proportion of the production originating directly or indirectly from the Banc; B. portion of the biomass by level of dependency of the various Ecopath groups to the Banc; C. current fishing mortalities (year^−1^) for these groups (B and C are referring to the base model only).

The degree of dependency is linked to trophic level (Figure 3). The highest proportion of the production originating from the Banc (60%) is observed around trophic level 2.3 (Figure 3a), mainly due to the groups large crustaceans and macrozooplankton especially abundant (and productive) in the Banc. The proportion decreases to 14% around trophic level 2.8, due to small pelagics (namely sardine and sardinelle abundant outside the Banc), and is close to 20% for all higher trophic levels. Here too, this proportion is not sensitive to the model assumptions, as estimates are similar for most trophic levels for all three models.

As a result of the high productivity originating from the Banc, a large part of the biomass present on the Mauritanian shelf is significantly dependent on the Banc (Figure 3b). Due to the structure of the model, the secondary producers are all either very dependent (>0.5) or independent (<0.1). In contrast, all groups with trophic level >3.5 show mostly intermediate dependency to the Banc ranging between 0.1 and 0.5. In other words, the primary and secondary production directly generated from the Banc progressively benefits all groups through the food web.

The fishing pressure exerted on the various groups differs according to their dependency to the Banc (Figure 3c). Groups considered as independent (dependency <0.1, namely sardine and mackerel), are subject to low fishing pressure (mean F< 0.2 year^−1^). Groups with a low dependency (0.1 to 0.3) are the most targeted ones, with average fishing mortalities around 0.3 year^−1^ for intermediate trophic levels (e.g. octopus, sardinelle, horse mackerel), and higher than 0.5 year^−1^ for the high trophic levels (e.g. large pelagics, groupers, large shelf fish). On average, fishing mortality for the most dependent groups (>0.3) is estimated at a low value (<0.1 year^−1^, for all trophic levels) but this result masks divergent trends among functional groups. The mean low value is due to the influence of the large biomass of small coastal fish that are not exploited and sparids, lightly exploited (C/B = 0.04). However, meagre is an important target species in the Banc and for the Russian pelagic fleet [51], and its fishing mortality was estimated at 0.42 in 2006 (EwE result, Table S9 in File S1). Medium-sized coastal fish were also submitted to large fishing mortality in 2006 (C/B = 0.49).

### Ecosim predictions 1991–2006

**Figure 4:**
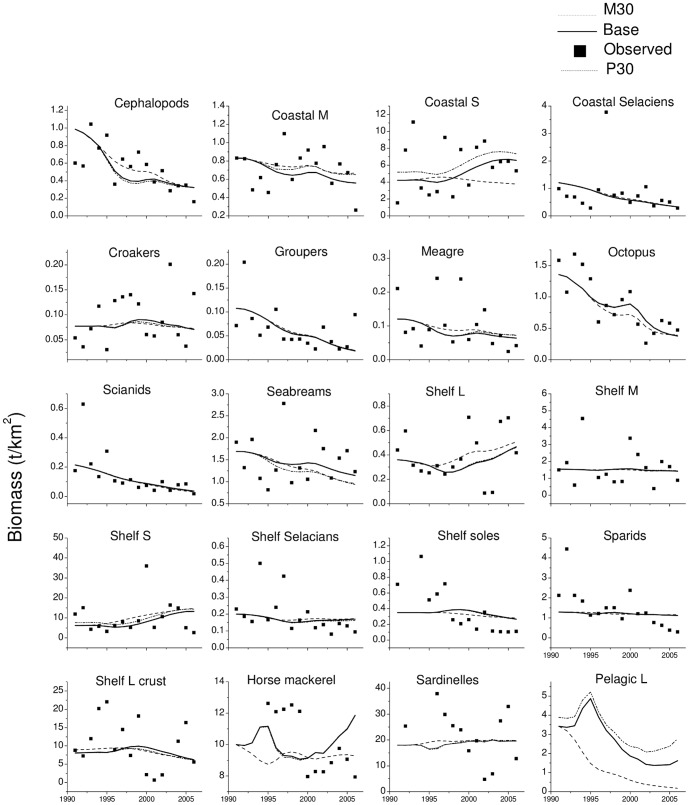
Observed and predicted biomass (t·km^−2^) for the period 1991–2006 using the base, M30 and P30 models. Except for Pelagic L, only groups with time series are shown.

The models (Base, P30 and M30) were able to predict the declining biomass trends observed for several demersal fish: coastal selacians, octopus, cephalopods, groupers, meagre, sciaenids, and seabreams (Figure 4). Shelf S biomass was predicted to increase in all models while coastal S biomass was not predicted to increase in the M30 model, but did so in the other two models in which vulnerabilities were estimated at high values (>100; Table S8 in File S1). Predictions for these two groups are driven solely by predation and suggest that the decrease in most of their predators' biomass caused the growth in population.

**Figure 5:**
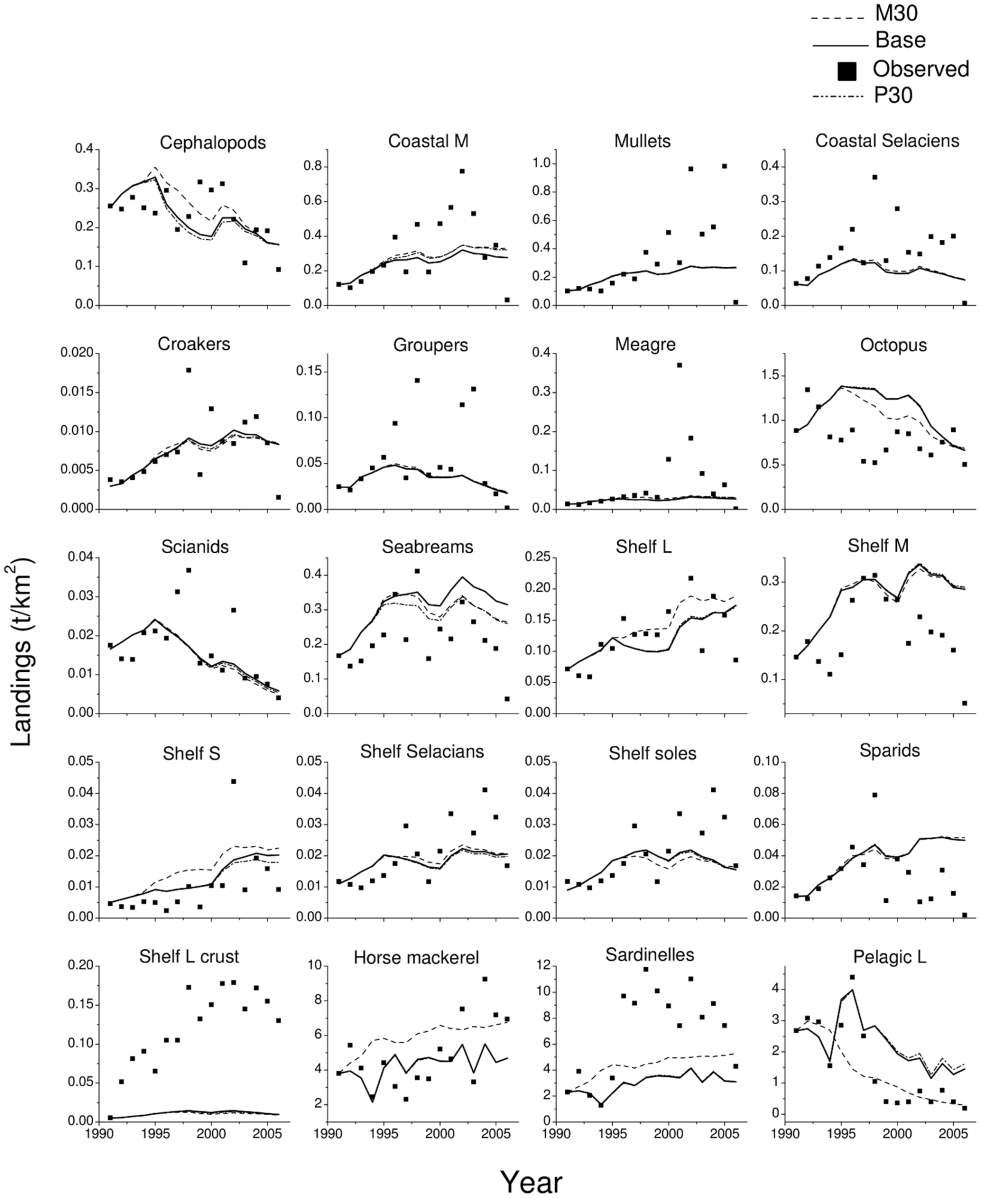
Observed and predicted catches for the period 1991–2006 using the Base, M30 and P30 models.

Trends in biomasses of sparids, croakers and shelf L crustaceans were not well predicted by the model: although increasing, the fishing pressure still remained a small part of total mortality at the final state of the simulation (i.e. 2006) and predation decreased over time. In addition, trends for coastal M and shelf soles are not well predicted as these groups are composed of a wide variety of species that may not be well sampled or equally vulnerable to the trawl. This combination of predation and fishing trends resulted in a stable simulated biomass rather than the observed decline. The group pelagic L is an interesting case as only catches were known. The three models estimated different vulnerability values and thus, different biomass trends, all declining. For the moment, there is no basis to decide which trend is the correct one and predictions for this group and small pelagics are unreliable. Sardinelles and horse mackerels are shown as examples of lack of fit to the highly variable trends in biomass and catches (Figure 4 and 5). In most cases the model predicts a decline in biomass during the study period for higher trophic level groups (23 out of 31), mostly predators. The exceptions are the coastal birds, small pelagic species, Shelf L, and small demersal fish on the shelf and in the Banc (Shelf S, Coastal S).

Although the development of effort by fleet does not account for changes in target species that may have occurred during the study period, the model predicted reasonably well the catches of several groups (e.g. cephalopods, croakers, shelf L) and especially so when the trends in biomass were well fitted and the trend was mainly due to fishing (Figure 5). For some groups (e.g. shelf soles, shelf L crustaceans and mullets) though, catches were underestimated as a result of badly estimated biomass.

**Figure 6:**
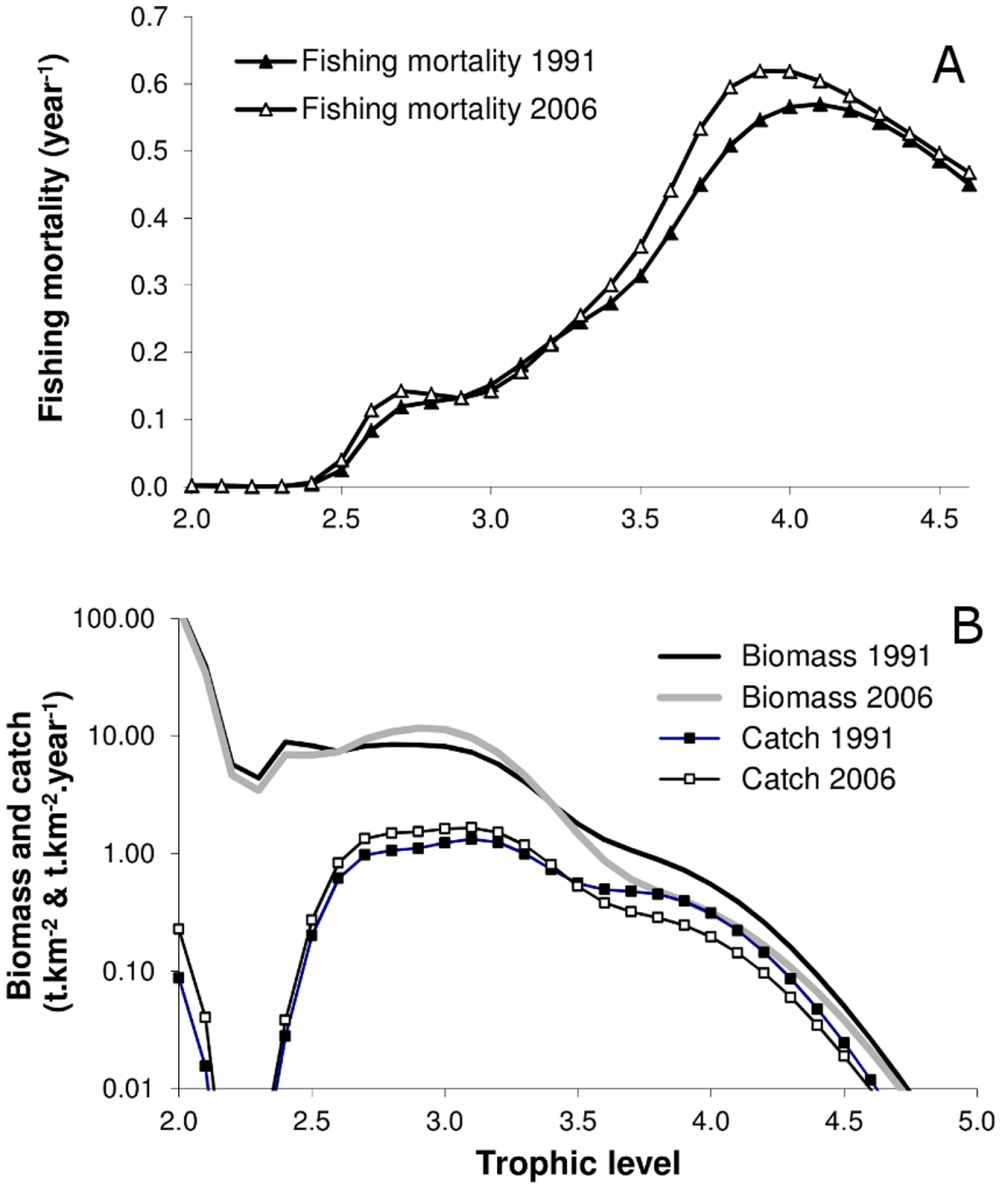
Change in the trophic spectra of A. fishing mortalities (year^−1^), B. biomass (t·km^−2^) and catch (t·km^−2^·year^−1^), between 1996 and 2006 (Base scenario). Note the logarithmic scale in panel B.

The trophic spectra provide a complementary and synthetic overview of changes that have occurred in the Mauritanian ecosystem between 1991 and 2006 (Figure 6). Fishing pressure, which appears moderate (<0.2 year^−1^) for intermediate trophic levels, and very high (>0.5 year^−1^) for high trophic levels, has slightly increased between the two periods, especially for the already heavily exploited predators (Figure 6a). By 2006, biomass of high trophic levels (>3.6) was halved while biomass of intermediate trophic levels (around 3) increased by almost 50% (Figure 6b). The increase in catch observed for mid-trophic-levels (small pelagics and demersal fish) is mainly due to an increase in their abundance, while the fishing pressure for these TLs remained almost the same. In 15 years, the mean trophic level of catches decreased by 0.1 while that of biomass decreased by 0.04.

### Simulations: the loss of habitat and the fictive fleet

**Figure 7:**
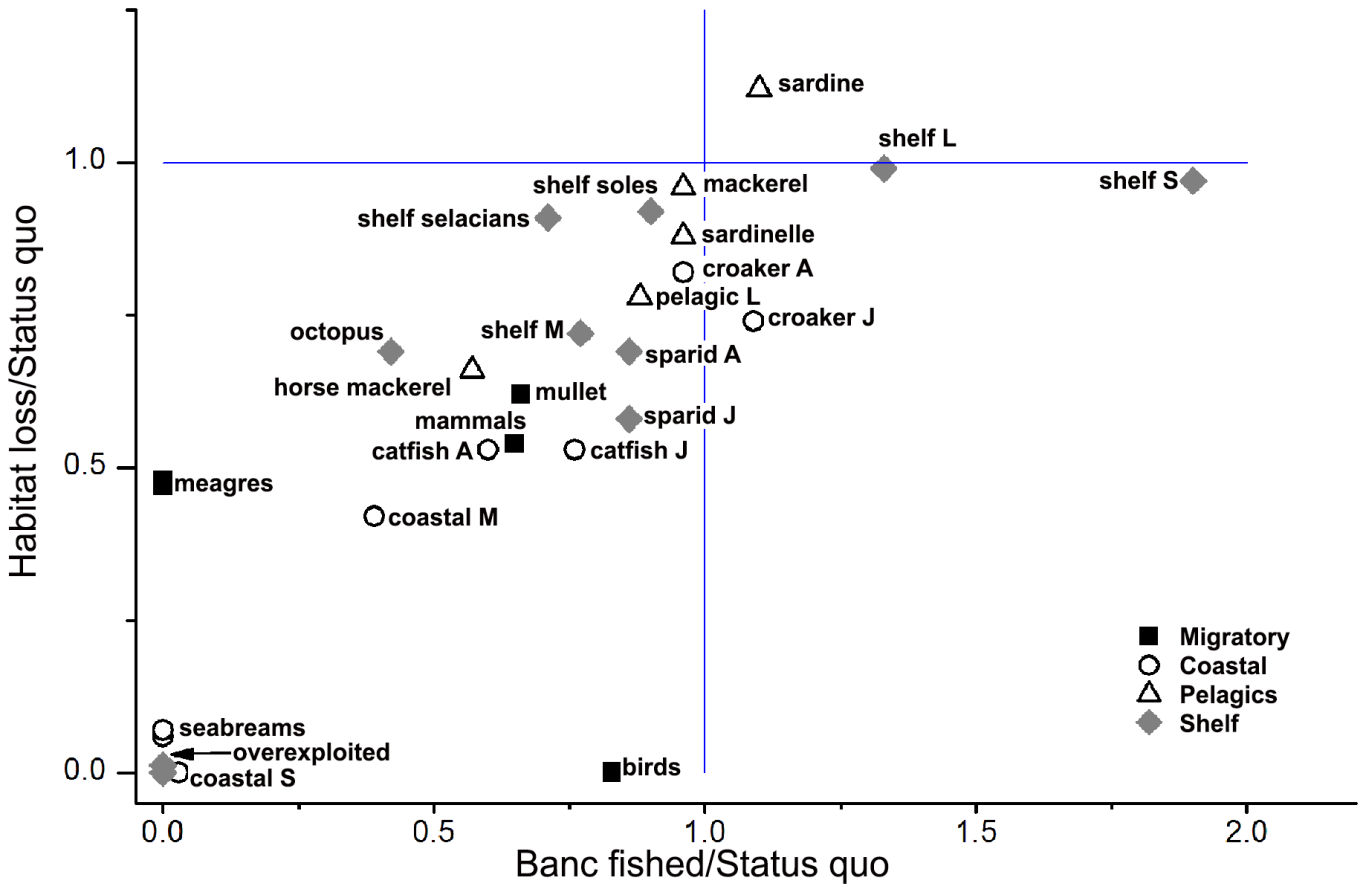
Relative biomass compared to the status quo under two scenarios: adding the fictive fleet in the Banc (B_fict_/B_Sq_ in x-axis) and the habitat loss in the Banc (B_Loss_/B_Sq_ in y-axis). A = adult, J =  juvenile.

The impact of the fictive fleet fishing in the Banc on any functional group varies depending on: 1. the group's initial trajectory (1991–2006); 2. its dependency on the Banc; and 3. its position in the food web, prey being often released from overexploited predators. Using the Base model, the biomass of sciaenids, coastal selacians, cephalopods, and both adult and juvenile of groupers, already falling at 50% or less of their initial biomass between 1991 and 2006, were predicted to decline further with the introduction of the fictive fleet, the biomass ratio falling below 1% (labeled “overfished” in Figure 7, *x* axis). Octopus, already overfished in 2006, is also predicted to be severely affected, but a bit less than the latter group (remaining biomass  = 42%) because the increased fishing mortality would be partly compensated by the release in predation from other cephalopods and sparids.

The fictive fleet would negatively affect several groups of demersal fish that were assumed to spend some time feeding in the Banc and thus, be available to the fleet. For instance, adult catfish, mullets, coastal M, and horse mackerels were predicted to decrease in biomass (remaining biomass 39 to 66%), while a strong decline close to extinction was predicted for coastal S, meagre and seabreams (Figure 7). The predicted decrease of marine mammals' biomass (65%) was caused by the decrease in cephalopods, their most important prey, while coastal birds, predicted to decrease (83%), would partly benefit from the release in competition for invertebrates. Shelf S benefited from the fictive fleet scenario as it was already increasing during the study period and its predators were predicted to decrease under the scenario conditions. Sardine and the juvenile croakers are also predicted to benefit from the decrease in predation.

A hypothetical loss of habitat in the Banc, causing the extinction of the primary and secondary production, would result in a decrease of biomass for most species except sardine. Compared to the status quo scenario, the remaining biomass would decrease to less than 1% for birds and the overexploited species (except for octopus) through the loss of their prey base (Figure 7, *y* axis). All juveniles would be severely impacted by the disappearance of the Banc's production. Groups such as catfish, octopus, meagre and croakers would also be impacted but this would be partly compensated by the decrease of their predators' biomass.

**Figure 8:**
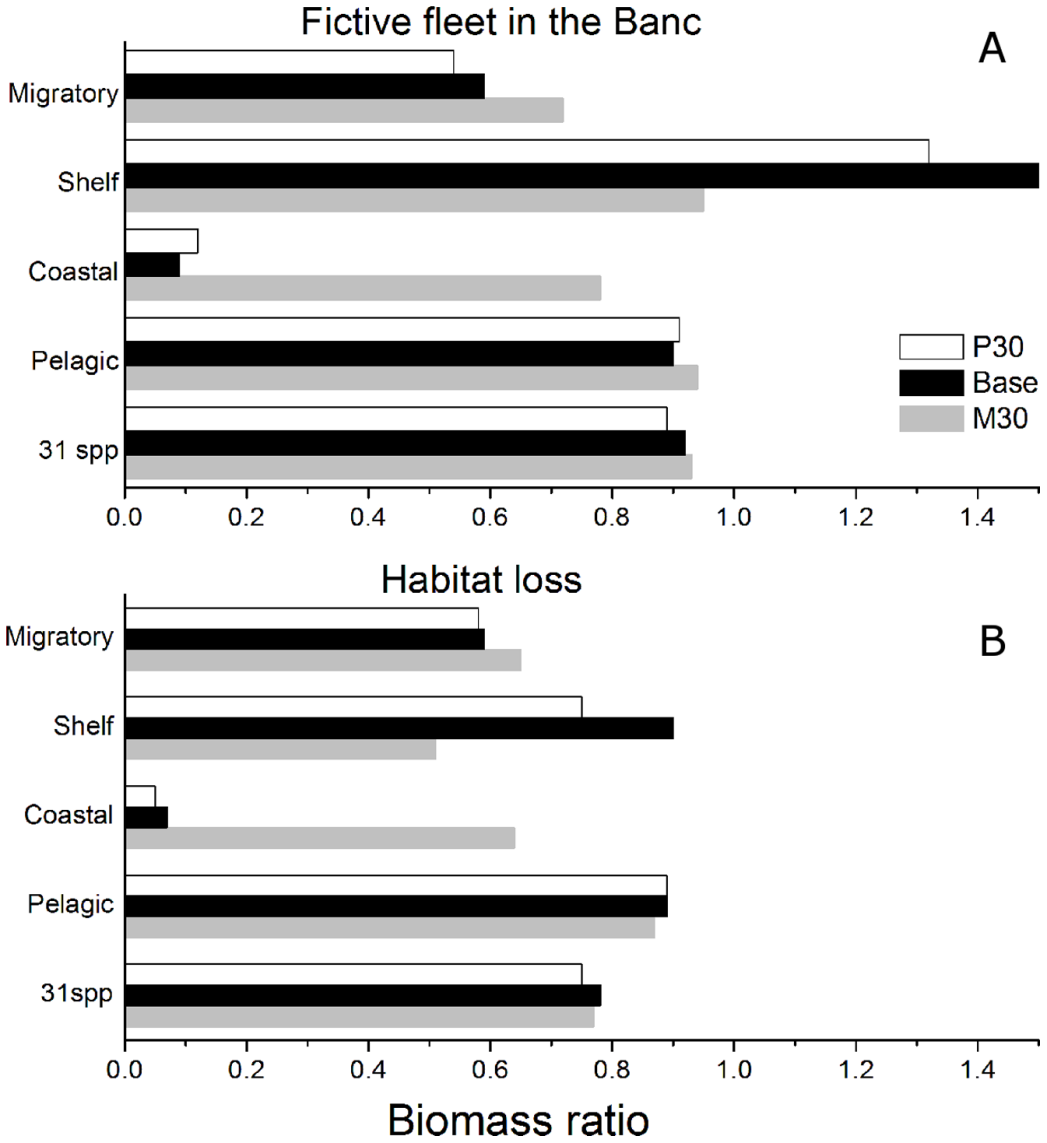
Biomass ratios for simulations involving an additional fictive fleet in the Banc (A) and the loss of the Banc as a habitat (B) for the three models (P30, Base, M30) by group of species (31spp, and coastal, pelagics and shelf functional groups).

**Figure 9:**
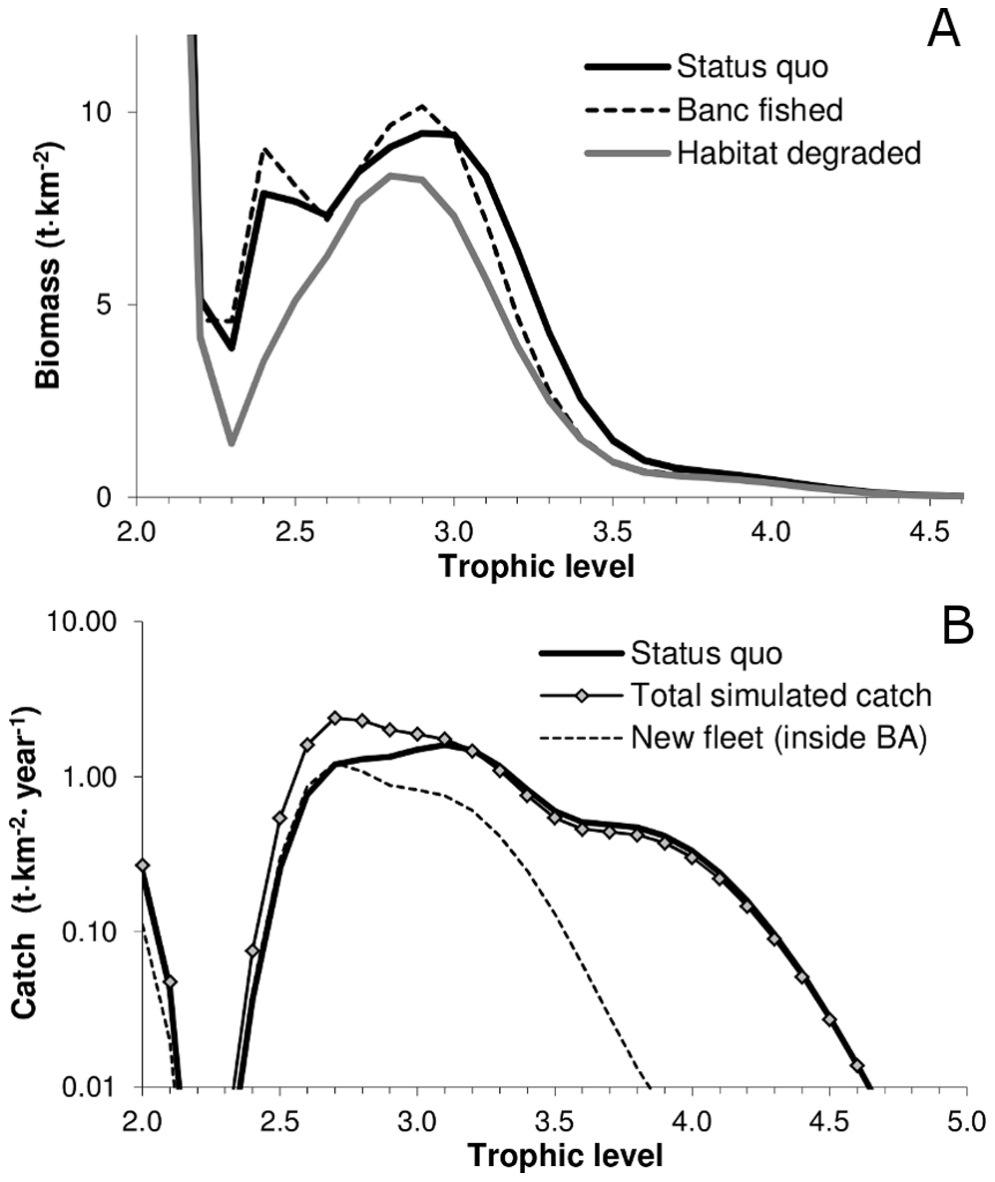
Biomass (A) and catch (B) spectra for the Base model and scenario of additional fishing (fictive fleet in the Banc and habitat loss).

The introduction of this fictive fleet (inside the Banc) is predicted to cause a decrease in biomass of the 31spp of higher trophic level (birds, fish, mammals and cephalopods) estimated at 8%, and this estimate varies little among scenarios (from 7 to 11%) (Figure 8a). Coastal groups, the most dependent on the Banc, were the most impacted, declining on average by 66% (22–91%), while shelf groups may benefit from a release in predation. Changes in abundance also differ depending on trophic levels (Figure 9a). Higher TL (>3.2) were predicted to decrease by 30% while lower levels increased by 5%. In comparison, the habitat destruction is predicted to result in a stronger decrease in biomass that affect a wider range of trophic levels due to direct destruction and the effects on predators. In this scenario, the remaining biomass of the 31 groups and of the coastal groups would decrease by 22% and 24% (36–93%) respectively (Figure 8b).

The fictive fleet would result in an increase in total catches estimated at 27% (from 15.2 to 19.3 t·km^−2^·year^−1^), mainly due to lower trophic level groups (Figure 9b) while catches of trophic levels >3.2 would decrease by around 9%. In other words, allowing a fishery inside the Banc would lead to a decrease in the mean trophic level of the catch. The new catch harvested within the Banc would amount to 7.6 t·km^−2^·year^−1^, but this would also lead to a 23% decrease in the catch originating from outside (and a stronger decrease for the catch of predators). These results suggest that limiting the fishery inside the Banc is currently supporting about a quarter of the catch taken from Mauritanian waters.

## Discussion

The model built for this study synthesized all available data from research surveys, fisheries and ecology for the Mauritanian EEZ and the Banc d’Arguin. Much uncertainty remains regarding pelagics as their biomass are poorly estimated and not much data on their links with plankton production and upwelling are available. Although the study area has been the subject of several ecological studies, there are still several notable gaps in diet composition (especially in the Banc), benthos and fish biomass in the Banc, and its importance as a nursery for instance. This study got around this difficulty by building three models that differed by the underlying hypotheses about the benthos biomass and the degree of reliance of each functional group on the Banc's planktonic and benthic invertebrates. In addition, simulations were designed to test the impact of different fishing rates and the loss of the Banc's primary and secondary production.

Although the model considers trophic relationships at the scale of the study site area, the spatial structure of species distribution and movements was partially taken into account by separating the sedentary species within and outside the Banc and grouping other species by their preferred habitats (coastal, shelf, migratory) in terms of depth gradient and possible use of the Banc. Perhaps more species depend on the Banc as juveniles, beyond the six functional groups considered in the model but it was not possible to include them at this stage. This means that our model only partially considers the functional role the Banc may have as nursery grounds for some species. Conversely our Ecopath approach, using spatially defined groups of primary and secondary producers, appears very powerful to analyse how organic matter produced within the Banc propagates through the food web, and to quantify the contribution of the Banc to the trophic functioning of the whole Mauritanian shelf ecosystem. The model shows that coastal species strongly depends on the production of the Banc. It also highlights the connections between coastal groups and those living on the shelf, and the links between the demersal and pelagic realms.

The present model differs from the preceding attempts by its emphasis on the possible connections between the Banc and the shelf as distinct habitats and the importance of the Banc for both the fishery and the ecosystem. This would not have been possible with previous models, dedicated to estimate the biomass of large predators in the Mauritanian EEZ [52], where the Banc was considered as a self-contained ecosystem [29], or the shelf was considered as a whole, ignoring the Banc's characteristics [28].

The three models show different dynamics for some groups, especially those with large uncertainties in the data (e.g. coastal S, pelagic L). As expected however, the predicted biomass and catches coincided better with observations for groups that exhibit clear trajectories related to fishing. Still, for this model as well as for any model, uncertainty about input values (biomass, diet, production) could influence the conclusions. For instance, the strong predatory effect of cephalopods on octopus (illustrated by the MTI) determines a good part of their biomass trends under various scenarios. Similarly, sparids' predation on cephalopods should be examined further. Also, groups such as selacians should be separated in demersal and pelagic species to avoid the super predator effect. Nevertheless, the results from the three scenarios differ mainly by the magnitude of the response to disturbance rather than the direction of the response, largely determined by the vulnerability to fishing and the state of exploitation rate and food dynamics (direct and indirect links).

The importance of the Banc in the ecosystem depends on the diet information and the assumptions made about their behaviour. As mentioned earlier, more information on benthos biomass and diet compositions at various times of the year, and locale could modify our findings. Nevertheless, the scenarios used cover a wide range of conditions that should be sufficient to give an idea of the magnitude of the variability involved. About 20% of production and catch of the 31 superior groups were estimated originating from the Banc and varied little with the model considered, the percentage reaching more than 50% for the coastal groups, highly dependent from the Banc. Yet, the simulated loss of production from the Banc led to a predicted 30% decline in the biomass for the higher level groups, while adding a fictive fleet in the Banc led to a reduction of almost 10%. This last simulation also showed that the current restriction of fishing enforced inside the Banc supports around a quarter of the catch currently harvested in Mauritania outside of the Banc. In contrast, smaller reserves were shown to have limited value for the ecosystems and target (sedentary) species while benefits to the fishery remain local (e.g. [19]).

The rapid increase in exploitation rates for high TLs over the last two decades resulted in a decrease in catch and biomass highlighting the overexploitation of these species. Such results confirm and generalize the diagnoses already established for several Mauritanian fish stocks, based on more usual stock assessment methods [35]. The increase in biomass of prey fish, while the biomass of top predators is decreasing, suggests a release in predation that might results from a top-down effect. Of course, the predictions concerning small pelagics are unsatisfactory at this stage as they are also driven by other factors (e.g. upwelling conditions) happening at a larger scale than the study area, and thus, it would be premature to consider that all ecological processes involved have been understood. The decline in mean TL coupled with signs of overexploitation of high TL species, and a decrease in their biomass and catch, is characteristic of a fishing down the food web process [53]. Since the catches on high TL species were not maintained at higher levels and there was no major change in target species (Figure 6a), the results do not suggest a fishing through the food web process [54].

Given the state of observed depletion of the main exploited groups (e.g., coastal selacians, seabreams, groupers) in 2006, the Banc did not likely grant protection against overfishing by controlling fishing mortality. This is not surprising given the relatively small amount of time spent in the Banc by their adult population and thus, the high vulnerability of most groups to fishing on the rest of the shelf. Single-species models have shown repeatedly that mobile species would not be protected if the closed area does not include a very large part of the fish geographic distribution and/or fishing effort is not controlled outside the closed area (see [18], [21]). Opening an additional fishery in the Banc, however, would augment the level of exploitation of the most vulnerable groups and cause further decline in their biomass. The loss of prey to fishing (e.g. S coastal fish) would contribute to further decline.

Trophic cascades resulting from fishing on large predators could be limited by movement dynamics causing predators and prey to move inside or outside the reserve as a response to perceived predation threat or feeding conditions [24], [25] Further analysis of the role of the Banc d’Arguin would include a formal spatially-structured model (e.g., Ecospace, the spatially structured component of Ecopath with Ecosim [55]) including more specific information on the fish movements and the biomass really present in the Banc. Such a model would also include the geographic location of each fleet, including the Imraguens' fishery in the Banc. In addition, species such as meagre and mullets are not well depicted in this model since a large part of their life cycle occurs within a large geographic area and would probably be better described, for fisheries management, using a spatially-structured model that encompasses the northwestern African coast and account for all national fisheries.

The main interest of our modelling approach was the ability to trace the production and showing the contribution of the reserve to the shelf ecosystem instead of on a single species. The importance of food web interactions was illustrated by the magnitude of the response to disturbance (fishing or habitat loss) that differed as a function of trophic level of species and changes in prey base, predation or competition level. The scenarios, illustrating contrasting management decisions, were useful to show the amplitude of the effects and offer a first attempt at understanding the ramifications on the entire ecosystem.

In the context of declining resources (this study and [32]) on the Mauritanian shelf, the pressure to open the Banc to industrial fishing is increasing. This study suggests that the Banc d’Arguin is an important source of production for the entire ecosystem and this role is more important than the catch that could be extracted from it. These findings are significant for fishing management in Mauritania because they support the current restriction on fishing in the Banc for the benefit of the biota and the fisheries occurring on the shelf outside the Banc.

## Supporting Information

File S1 Text S1. Table S2, Diet composition for the base model. Table S3, Effort used for each fleet. Table S4, Composition of the marine mammal group and parameters values for biomass, and P/B and Q/B ratios. Table S5, Composition of the coastal bird groups and parameters used in the model. Table S6, Parameters used to calculate benthos biomass and production by unit of biomass (P/B). Table S7, List of biomass and catch time series available and used in the Ecosim fitting process. Table S8, Proportion of invertebrates from the Banc (pBAi) in diets imposed in each model (M30, Base and P30), and resulting biomass, ecotrophic efficiency (EE) from balancing the Ecopath model, and vulnerability values for each Ecosim model (M30, Base and P30) fitted to the time series. Table S9, Biomass, catch and fishing mortality (C/B) by functional groups based on EwE data (1991) and estimations (2006). Figure S1, Simplified food web structure showing only the trophic links for which the absolute value of the MTI impact is higher than 0.1.
(DOCX)
