# Supplementary material for: Assessing the Contribution of Marine Protected Areas to the Trophic Functioning of Ecosystems: A Model for the Banc d’Arguin and the Mauritanian Shelf
Source: PLoS One. 2014 Apr 11;9(4):e94742. doi: 10.1371/journal.pone.0094742 (PMC3984206; doi:10.1371/journal.pone.0094742)
Supplement: File S1 — Text S1. Table S2, Diet composition for the base model. Table S3, Effort used for each fleet. Table S4, Composition of the marine mammal group and parameters values for biomass, and P/B and Q/B ratios. Table S5, Composition of the coastal bird groups and parameters used in the model. Table S6, Parameters used to calculate benthos biomass and production by unit of biomass (P/B). Table S7, List of biomass and catch time series available and used in the Ecosim fitting process. Table S8, Proportion of invertebrates from the Banc (pBAi) in diets imposed in each model (M30, Base and P30), and resulting biomass, ecotrophic efficiency (EE) from balancing the Ecopath model, and vulnerability values for each Ecosim model (M30, Base and P30) fitted to the time series. Table S9, Biomass, catch and fishing mortality (C/B) by functional groups based on EwE data (1991) and estimations (2006). Figure S1, Simplified food web structure showing only the trophic links for which the absolute value of the MTI impact is higher than 0.1. (DOCX) [file pone.0094742.s001.docx]

Supplementary material

# Text S1

## The model

Ecosim is a tool for dynamic simulations based on the Ecopath model, an instantaneous image of the ecosystem at a given time [[1-3](#_ENREF_1)]. Ecopath models account for the biomass of each functional group of species, their diet composition, production per unit of biomass (P/B), consumption per unit of biomass (Q/B), mortality from predators and fishing, accumulation of biomass and net migration. The principle behind this ecosystem modelling approach is that, on a yearly basis, biomass and energy are conserved. The proportion of the mortality of each group that is accounted by the model (fishing, predation, biomass accumulation, migration) is called ecotrophic efficiency (EE). The P/B is considered equal to total mortality under equilibrium condition [[4](#_ENREF_4)] since production and losses would be equal. The total mortality (Z) is thus computed as the sum of fishing mortality (F) and natural mortality (M) which includes mortality by predation.

Ecosim uses a system of differential equations to describe changes in biomass and flows with the system by accounting for change in predation and fishing

eq. 1

where gi is the net growth efficiency; Q_ji_ and Q_ij_ are the consumption rate of group *j* by group *i* and the consumption of group *i* by group *j* respectively; I_i_ the immigration in t/km^2^; M_0,i_ the instantaneous non-predatory natural mortality; F_i_ the fishing mortality; and e_i_ the immigration rate. Ecosim can incorporate the multiple life history stages (stanzas) for species having ontogenic changes in trophic and habitat preferences. The stanzas are linked and their respective production and consumption per unit of biomass (P/B_i_ and Q/B_i_ respectively, per year), and growth are calculated from a baseline estimate for the leading group (the adults in our case). It is assumed that growth for each stanza follows a von Bertalanffy growth curve, and that the survivorship is stable through ages.

The estimation of consumption of prey *i* by predator *j* (Q_ij_) at each time step is based on the foraging arena theory [[2](#_ENREF_2),[5](#_ENREF_5)] and calculated as:

eq. 2

where a_ij_ is the rate of effective search for prey *i*, *v* (vulnerability) is the rate of exchange between the vulnerable and invulnerable prey biomass pools. The estimate of a_ij_ is obtained by solving equation 2 using parameters from the Ecopath model and conditional on the value of *v* (default value=2). Low vulnerability (1> v_ij_<1.5) implies a donor-control or type II functional response, while a large value implies that a change in biomass of the predator will cause a corresponding change in the mortality rate of its prey. Model fitting is achieved by estimating vulnerability values that minimizes the sum of squares of differences between model predictions and times series of biomass and catch.

## Data

Note: Except for the groups designating fish of a particular family or one species, fish groups names are typically composed of first, their habitat preference (coastal, pelagic, shelf), and second, their size (small (S), medium (M) or large (L)) (Table S1).

### Fish

Biomasses of demersal fish, octopus and cephalopods were derived from the annual trawl survey and estimated using a delta general linear model (delta-GLM) accounting for strata, year, and catchability coefficients for groups of taxa, following the method described in [[6](#_ENREF_6),[7](#_ENREF_7)]. Pelagic fish biomass were obtained from yearly acoustic surveys estimates carried out by the Norwegian vessel N/O Dr. Fridtjof Nansen, covering the north-western coast of Africa (region) starting before 1995, and the vessel El Awam, covering the Mauritanian coast starting in 2003. The biomass of sardine and mackerel, available only at the scale of the region, were downscaled assuming that the biomass present in Mauritania was proportional to the ratio of Mauritanian catch to that of the whole region: Bmaur=Bregion*Cmaur/Cregion. In addition, the biomass of horse mackerel estimated from the Norwegian vessel for Mauritania was downscaled using the average biomass estimate from the Mauritanian survey. Such estimates are potentially unreliable given the variable migrations linked to variations in upwelling strength. Consequently, the time series of biomass of mackerel, sardine, and anchovy were considered doubtful and non-representative, and were not used to fitting the model. The (negative) biomass accumulation rates (/year) were calculated from the biomass trends observed for the various exploited groups before 1991 (see [[6](#_ENREF_6)]) for fish and cephalopods (Table 1)

The residence time of migratory species in the study area was derived from knowledge of their migrations and timing of their fishery. Small pelagics were generally present 8 months of the year [[8](#_ENREF_8)], meagre 6 months [[9](#_ENREF_9)] and mullets 5 months [[10](#_ENREF_10)]. Residence times were used to scale down the biomass present in the study area. Biomasses of fish present in the Banc d’Arguin were estimated from the trawl surveys Amrigue in 2000-2007. These estimates, although incomplete and probably biased because of the gear limitation in such a coastal environment, were added to the shelf biomass estimate but they are often insignificant compared to estimates for the rest of the shelf.

Fish Q/B values were preferably estimated with the empirical equation from [[11](#_ENREF_11)]:

log10(Q/B) = 7.964 - 0.204log10(W∞) – 1.965T' + 0.083A + 0.532h + 0.398d eq. 3

where Q/B is the yearly food consumption of a fish population as a percentage of its biomass, W∞, the mean asymptotic (or maximum) weight (g) of the fish in the population in question, T’ = 1000/Kelvin (Kelvin = °C + 273.15) is the mean habitat temperature, and d (=1 for detritivores), carnivores being identified when both h and d equal 0, A=aspect ratio of the tail. An alternate estimate was obtained with:

Q/B =106.37*0.0313(1000/273.1+T) * W∞-0.168*1.38Pf*1.89Hd [[12](#_ENREF_12)] eq. 4

where the food type (Pf =1 for carnivores and Hd =1 for detritivores and herbivores). We preferred the results of equation 3 as equation 4 generally produces higher (too high) Q/B estimates. For selacians and fish for which we did not have sufficient information, the ratio P/Q was fixed at 0.15 and 0.2 respectively.

Natural mortality (M) of fish was derived from the empirical model of Pauly [[13](#_ENREF_13)]:

log10(M)=0.333-0.246*Log10(L∞)+0.744*Log10(K)+0.01*T eq. 5

where K and L∞ (cm) refer to the curvature and asymptotic (total) length parameters of the von Bertalanffy growth function, and T is the mean annual water temperature in Celsius (19.5 °C). Alternative estimates were taken from Hoenig’s empirical equation [[14](#_ENREF_14)] for species for which the longevity is available:

ln(M)=1.44-0.982*ln(max age) eq. 6

using the maximal observed age found in Fishbase. The lower of the two estimates was kept.

Fish diets were preferably derived from studies from the region or elsewhere in similar habitats, as found in the literature and in Fishbase, and local qualitative information from local fisheries biologists (Table S2). The composition of each fish groups is listed in Table S1.

### Fishing data

Catches for the artisanal and the industrial (demersal and pelagic) fleets were obtained from the Mauritanian database. There is no information on discards for any of the fleets. Catches classified as ‘other fish’ were allocated to the groups Coastal M and Shelf M (25% each) and the remaining 50% to the other demersal fish in proportion to the amount already reported. Catches for small pelagics, octopus (*Octopus vulgaris*) and ethmalosa (*Ethmalosa fimbriata*, group coastal M) were obtained from the estimates of the Fishery Committee for the Eastern Central Atlantic Working Group [[15](#_ENREF_15)]. Effort trends consisted in the number of vessels for artisanal and industrial demersal fleets [[6](#_ENREF_6)] while the average gross registered tonnage (GRT) was used for the pelagic fleet [[16](#_ENREF_16)]. During the study period, effort increased 5 times for the artisanal fleet and 2 times for industrial pelagic and demersal fleets (Table S3).

### Mammals and birds

The marine mammal group includes only species mostly feeding in the study area, ignoring oceanic species that feed marginally in the area [[17](#_ENREF_17)] (Table S4). Monk seals biomass (*Monachus monachus*) was taken from a previous model of the Mauritanian coast [[18](#_ENREF_18)]. Biomasses for other marine mammals were obtained from the density estimates derived from global databases and habitat characteristics for the north-western African coast [[17](#_ENREF_17)], assuming similar densities in Mauritania as in the region, and Q/B ratios were calculated using an empirical equation based on body weight [[19](#_ENREF_19)] (Table S4). P/B ratios were set at 0.02 /year for killer whales, 0.036 for beaked whales [[19](#_ENREF_19)] and 0.05 for all other species collectively [[17](#_ENREF_17)] (Table S4). Diet compositions were obtained from [[20](#_ENREF_20)] and completed with qualitative information found in other publications [[21](#_ENREF_21),[22](#_ENREF_22)]. There are no time series for birds and mammals.

Coastal birds are either breeders or migratory species spending part of the year in Mauritania, feeding in coastal waters or on muddy flats in the Banc d’Arguin (Table S5). The abundance of breeding birds was calculated from counts of breeding pairs assuming that the total population amounted to 3 times the breeding pairs [[23](#_ENREF_23)]. Waders were counted in the winter in the Baie d’Arguin, an area carrying about the same density of waders as the entire Banc but 18 times smaller [[24](#_ENREF_24)]. The authors estimated the possible numbers of birds in the Baie at 1.4 to 2 fold the actual counts. Hence, the number of waders counted in the Baie was multiplied by 25.5 to obtain the abundance for the Banc d’Arguin (Table S5). The ration in kg/day was estimated from an empirical equation [[25](#_ENREF_25)]. Diet compositions were derived from both qualitative and quantitative studies found in the literature. Natural mortality was obtained from compilations found in the literature for 7 species [[26-28](#_ENREF_26)]. The resulting natural mortality (M, 0.28/year) is consistent with a weighted average maximal age of 17 years for the 20 species for which the information was available.

### Cephalopods

Cephalopods were divided in two groups, octopus and other cephalopods. Octopus mortality was estimated at 0.1/month for a life span of about 1.5 year and adults dying massively after spawning [[29](#_ENREF_29)]. Thus, M was estimated at 1.2 /year and F at 0.64 based on the ratio C/B. Q/B was estimated by the model using a P/Q value of 0.3. Other cephalopods were assumed to have similar natural mortality and P/Q ratio as octopus, while fishing mortality (L/B) amounted to 0.43. Diet compositions were derived from qualitative and quantitative studies for *Octopus vulgaris* [[30](#_ENREF_30)], *Loligo forbesi* and *L. vulgaris* [[31-33](#_ENREF_31)] and *Illex illecebrosus* [[34](#_ENREF_34)].

### Plankton

Zooplankton on the shelf was obtained from transect sampling outside the Banc d’Arguin, which means covering the northern part of the ZEE [[35](#_ENREF_35)]. The mesozooplankton comprises copepods, cladocerans, gastropod and lamellibranch larvae, dinoflagellates, etc. Their biomass is estimated at 68 t/km^2^ using an average depth of 66 m, conversion ratio AFDW:DW of 0.904:1 and DW:WW 1:0.186 from [[36](#_ENREF_36)], and assuming that densities are similar in the southern part of the ZEE. The macrozooplankton includes euphausids, mysids, chaetognaths, salps, hydrozoa, fish larvae, etc. Their biomass was estimated at 4.21 t/km^2^ using taxa specific conversion ratios from AFDW to WW [[36](#_ENREF_36)]. There was no information for zooplankton in the Banc, so these biomasses were left for the model to estimate using an EE of 0.8. P/B and Q/B values for mesozooplankton were set at 24 /year and 112 /year based on [[37](#_ENREF_37)]. This value of P/B is lower than estimates compiled for *Pseudocalanus* in various north hemisphere seas (52 (32-73) per year in [[38](#_ENREF_38)]). Herbivorous macrozooplankton were estimated to ingest a maximum of about a third of their body weight per day [[35](#_ENREF_35)] or 110 /year. P/B and Q/B values for macrozooplankton were set at 4.3 and 17 based on a compilation of estimates for northern seas [[39](#_ENREF_39)]. Mesozooplankton were assumed to feed mainly on phytoplankton while macrozooplankton’s diet was based on qualitative information found in the literature (see [[39](#_ENREF_39)]).

### Benthic fauna

The biomasses of shelf macrobenthos were obtained from two transects (north and south) estimates crossing the shelf from the Banc to the limit of the shelf [[40](#_ENREF_40)], using taxa specific conversion ratios from AFDW to WW [[36](#_ENREF_36)]. Polychaetes were the main group found throughout the plateau and no gradient in biomass was found with depth. The authors attributed the higher biomass resulting from their study compared with that of the southern shelf found in the literature to the effect of the high productivity around the upwelling in the north (Cape Blanc).

In the Banc d’Arguin, the biomass of benthic invertebrates was estimated only for tidal flats that spans about 500 km^2^ [[41](#_ENREF_41)]. Shrimps, sampled with push nets in both the intertidal and subtidal areas of the Banc, may be underestimated as the area sampled is small and small shrimps were not included in the analysis [[42](#_ENREF_42)]. Densities of shrimps, molluscs and worms were estimated by assuming that the biomass outside the subtidal habitats was equal to that of the intertidal (Table S6). The violonist crab (*Uca tangeri*) and the gastropod *Senilia senilis* were both assumed to be restricted to tidal flats. Small crustaceans (amphipods, isopods, etc) were thought to be underestimated given the sampling gear and thus their density was assumed to be a minimal estimate for the entire area of the Banc. As a first approximation, meiobenthos biomass was assumed to amount to about 6% of the macrobenthos biomass, the observed proportion for the shelf, but their biomass was increased to balance the model. P/B values were obtained from the literature [[43-45](#_ENREF_43)] and P/Q values (=gross efficiency) from [[43](#_ENREF_43)].

### Primary production

Phytoplankton species composition in the Banc d’Arguin is different from that of the upwelling area where diatoms are abundant [[46](#_ENREF_46)]. Crude estimates of phytoplankton biomass for the Banc d’Arguin were obtained from 2 studies, one covering the Baie du Lévrier in 1973 and the other the inshore waters [[46](#_ENREF_46)]. The productivity in mgC/m^2^/d were converted in wet weight using a C:WW ratio of 1:9 [[47](#_ENREF_47)] and assuming a P/B of 100. The average of these two estimates amounted to 31 t/km^2^ in the Banc, ignoring the very low biomass on tidal flats [[24](#_ENREF_24)]. The primary production for the shelf, 2,549 mgC/m^2^/day, was derived from SeaWifs and obtained from the Sea Around Us project web site (http://www.seaaroundus.org/eez/478.aspx). Using the same C:WW conversion ratio, the biomass amounted to 83.7 t/km^2^.

The biomass of seagrass beds of tidal and subtidal habitats was derived from Vermaat et al. [[48](#_ENREF_48)] using DW:WW ratio of 0.199:1 [[36](#_ENREF_36)] for a total of 1707 g/m^2^ in the Banc. The Banc covers 43% of the littoral area between 5 and 20 m deep and seagrass beds outside the Banc were assumed to be half as dense as in the Banc. Thus, the Banc was assumed to contain 72% of the seagrass biomass. The average above ground production was estimated at 2 AFDW g/m^2^/d, or 6921 gWW/yr using the same ratios as above, resulting in a P/B of 4.1/year.

## Balancing and fitting the model

Using the input values, Ecopath solves simultaneous linear equations and estimates the missing parameters, often the Ecotrophic Efficiency (EE) value. The balancing process is done manually by checking inconsistencies in data, adjusting biomasses, P/B ratio, and diet composition, starting with parameters that were deemed less reliable. As such, diet compositions are often modified on the account of seasonal and individual variation and sampling error. Overestimates of the proportion of rare prey in the diet of an abundant predator is a common source of excessive mortality. P/B values are often overestimated and the P/Q ratio is sometimes too low because of Q/B overestimates (e.g. [[39](#_ENREF_39)]).

Time series of biomass, catch and fishing effort by fleet were used to fit Ecosim temporal simulations for the period 1991-2006. The model was driven by fishing effort (Table S3) and fit to the observed biomass and catch (Listed in Table S7). The fitting occurred in two recursive steps aiming at decreasing the sum of squares between the predicted and observed biomass and catch. The model was first explored to identify the most sensitive predator-prey relationships and second, the vulnerability values were estimated using the formal non-linear search procedure. We used the knowledge gained in the manual fitting to start the search procedure with different initial parameters to avoid being caught in local optimal solutions.

In addition, the biomass time series of mackerel, catfish and sardine were not considered (weight=0 on a scale of 0 to 1) to fit the model because their time series were not deemed representative of the population trends. The biomass time series of coastal S and shelf L crustaceans were given a weight of 0.5 because of their large variability and the little number of species the time series covered compared with the number of species included in these groups. The search for vulnerability values was carried out only for functional groups with time series, excluding sardines.

The upwelling indices for the study period was obtained from IMROP (Institut Mauritanien de Recherches Océanographiques et des Pêches) and used to modify the production of the shelf phytoplankton group.

## Balancing and fitting results

Balancing this model required to change the diet of several groups. Predation had to be decreased on most juveniles groups, Coastal M, cephalopods, shelf soles, and the 2 groups of macrozooplankton. The predation was transferred in large part to adult groups, sardinelles, sardine, shelf S, coastal S, and the 2 groups of worms and mesozooplankton. The values of P/Q was low for several pelagic species and thus the P/Q ratio was fixed at 0.2, leaving Ecopath to estimate a more appropriate value of Q/B for sardine, horse mackerel and mackerel. The biomass of shelf soles, octopus, BA meiobenthos and BA crustaceans were increased. The biomass of sardinelles was obviously too large and the EE too small so the biomass was decreased from 24 to 18 t/km^2^.

Several explorative trials at fitting the model to time series running from 1991 to 2006 led to the conclusion that the index of upwelling did not improve the fit to demersal or pelagic species. Changes in primary production induced by the index of upwelling introduced more noise in biomass trends for demersals (except for octopus), increasing the sums of squares for these groups. The observed biomass trends of sardine, sardinelles, mackerel, and horse mackerel exhibit different trajectories during the study period as they are function of several factors such as the strength of the upwelling, physical conditions and their impact on plankton dynamics, and fish habitat preferences [[8](#_ENREF_8)]. These signals can also be confounded by changes in the position of their geographical centre of gravity. For instance, sardines increased off the Western Sahara (north of Mauritania) as a result of a southward displacement of their centre of distribution in the 1970s [[8](#_ENREF_8)]. Finally, the upwelling index for the study period is rather flat, except for increases in 1991-1994 and 1997-1998. Hence, it is not surprising that a model with no detail on phytoplankton and mesozooplankton cannot resolve the dynamics of each small pelagic species. Our objectives being to assess the role of the Banc d’Arguin, we did not attempt to fit the small pelagic component trends any further, expecting the model to explain mostly the dynamics of the demersal component. Fitting was improved by taking into account the negative biomass accumulation (except for shelf soles). P/B ratios had to be decreased for hake, octopus, cephalopods. The sum of squares of the fitted model is 280 compared to the initial 423 when all vulnerability values were set at 2.

## Mixed trophic impact (MTI)

Direct and indirect interactions within the ecosystem were analyzed using the Mixed Trophic Impact (MTI) routine of Ecopath, which assesses the relative impact of a slight increase in abundance of any group on the biomass of other groups in the food web [[2](#_ENREF_2)]. The MTI index, scaled from -1 to 1, was calculated for every group of the model. The net cumulative impact Qij of a predator j on a prey i is calculated as:

qji= DCji –Qji/Qj

where DCji is the the proportion of the prey *i* in the predator’s diet (the positive effect of the prey), while Qji/Qj is the negative effect, Qji is the quantity eaten by predator *j* of prey *i* and Qj is the total consumption by predator *j*. To this direct impact are added all indirect impacts of the predator on the prey calculated as the sum of all indirect paths calculated as the product of the Qij values in each paths [[1](#_ENREF_1),[49](#_ENREF_49)]. Table S1. Composition of fish functional groups.

| N | Group | Species |
| --- | --- | --- |
| 3 | Meagre | *Argyrosomus regius* |
| 5 | Mullets | *Mugil cephalus;Mugil capurrii; Liza aurata; Liza falcipinnis; Liza ramada; Mugil cephalus; Liza dumerili* |
| 6 | Pelagic L | *Orcynopsis unicolor; Euthynnus alletteratus; Sarda sarda; Auxis thazard thazard; Auxis rochei rochei; Pomatomus saltatrix; Trichiurus lepturus; Lepidopus caudatus; Scomberomorus tritor; Mola mola; Sphyraena guachancho; Sphyraena sphyraena; Lichia amia; Coryphaena hippurus; Elops lacerta* |
| 7 | Mackerel | *Scomber japonicus* |
| 8 | Sardine | *Sardina pilchardus* |
| 9 | Sardinelles | *Sardinella maderensis; Sardinella aurita; Engraulis encrasicolus* |
| 10 | Horse mackerels | *Trachurus trachurus; Trachurus trecae; Caranx rhonchus* |
| 11 | Coastal selacians | *Dasyatis centroura; Dasyatis pastinaca; Gymnura altavela; Leptocharias smithii; Mustelus mustelus; Myliobatis aquila; Pteromylaeus bovinus; Rhinobatos cemiculus; Rhizoprionodon acutus; Torpedo marmorata; Zanobatus schoenleinii; Rhinobatos rhinobatos; Dasyatis margarita; Dasyatis margarita; Raja miraletus; Rhinoptera bonasus; Rhinoptera marginata; Dasyatis marmorata* |
| 12 | Coastal M | *Solea solea; Cynoglossus senegalensis; Solea senegalensis; Cynoglossus canariensis; Pegusa lascaris; Cynoglossus monodi; Synaptura lusitanica; Synaptura lusitanica nigromaculata; Synaptura cadenatii; Pegusa triophthalma; Psettodes belcheri; Psettodes bennettii; Dicentrarchus punctatus; Aluterus schoepfii; Balistes punctatus; Balistes carolinensis; Parapristipoma octolineatum; Galeoides decadactylus; Pentanemus quinquarius; Mullus barbatus; Eucinostomus melanopterus; Drepane africana; Scorpaena elongata; Ethmalosa fimbriata; Sarotherodon melanotheron melanotheron; Brachydeuterus auritus; Stephanolepis hispidus; Lethrinus atlanticus; Chaetodipterus lippei; Syngnatus typhle; Spaeroides marmoratus; Sphoeroides spengleri* |
| 13 | Coastal S | *Gobius niger; Pomatischistus marmoratus; Pomatoschistus microps; Solitas gruveli; Atherina lopeziana* |
| 14-15 | Croakers | *Pseudotolithus senegallus; Pseudotolithus typus; Pseudotolithus senegalensis* |
| 16-17 | Seabreams | *Plectorhinchus mediterraneus ; Sparus aurata; Diplodus puntazzo; Diplodus sargus sargus; Diplodus sargus cadenati; Diplodus vulgaris; Diplodus bellottii; Dentex canariensis; Dentex gibbosus; Pagrus pagrus; Pagrus caeruleostictus; Pagrus auriga; Pagellus bogaraveo; Pagrus pagrus; Lithognathus mormyrus* |
| 18-19 | Catfish | *Arius heudelotii; Arius latiscutatus; Arius parkii* |
| 20 | Shelf selacians | *Galeorhinus galeus; Paragaleus pectoralis; Raja undulata; Oxynotus centrina; Raja straeleni; Squalus blainville; Symphodus bailloni; Sphyrna lewini; Sphyrna zygaena; Taeniura grabata; Torpedo torpedo; Raja clavata; Rostroraja alba; Galeus polli; Heptranchias perlo; Squatina aculeata; Torpedo nobiliana* |
| 21 | Shelf L | *Muraena helena; Conger conger; Lagocephalus laevigatus; Fistularia tabacaria; Dicentrarchus labrax; Brotula barbata; Zenopsis conchifer; Zeus faber; Ruvettus pretiosus; Malacocephalus laevis; Lophius budegassa; Lophius piscatorius* |
| 22 | Shelf M | *Halobatrachus didactylus; Ephippion guttifer; Pseudupeneus prayensis; Chelidonichthys lucerna; Aspitrigla cuculus; Trachinocephalus myops; Uranoscopus polli; Pomadasys incisus; Pomadasys jubelini; Pomadasys rogerii; Dactylopterus volitans; Acanthurus monroviae; Callionymus lyra; Mullus surmuletus; Pomadasys perotaei; Branchiostegus semifasciatus; Scorpaena scrofa; Gephyroberyx darwinii; Pontinus accraensis; Batrachoides didactylus ;Elops senegalensis; Chloroscombrus chrysurus; Alectis alexandrinus; Selene dorsalis; Campogramma glaycos; Stromateus fiatola; Caranx crysos; Caranx senegallus; Trachinotus ovatus; Seriola carpenteri; Serranus cabrilla; Cepola pauciradiata; Scarus hoefleri; Pontinus kuhlii; Helicolenus dactylopterus dactylopterus; Scorpaena stephanica* |
| 23-24 | Groupers | *Epinephelus aeneus; Epinephelus caninus; Epinephelus costae; Epinephelus goreensis; Epinephelus marginatus; Epinephelus itajara; Epinephelus fasciatus; Mycteroperca rubra; Rypticus saponaceus* |
| 25-26 | Sparids | *Pagellus acarne; Pagellus bellottii bellottii; Dentex maroccanus; Dentex angolensis; Dentex macrophthalmus; Boops boops; Sarpa salpa; Spondyliosoma cantharus* |
| 27 | Scianids | *Sciaena umbra; Umbrina canariensis* |
| 28 | Shelf soles | *Arnoglossus imperialis; Arnoglossus capensis; Bothus podas; Syacium guineensis; Citharus linguatula; Microchirus boscanion; Monochirus hispidus; Bathysolea polli; Arnoglossus laterna; Dicologlossa cuneata* |
| 29 | Shelf S | *Pterothrissus belloci; Antennarius striatus; Bembrops heterurus; Saurida brasiliensis; Serranus scriba; Trachinus armatus; Anthias anthias; Capros aper; Chaetodon hoefleri; Trigloporus lastoviza; Chelidonichthys obscurus; Chilomycterus spinosus mauretanicus; Chromis chromis; Lepidotrigla cadmani; Xyrichtys novacula; Macroramphosus scolopax; Chlorophthalmus agassizi; Scorpaena notata; Scorpaena angolensis; Scorpaena laevis; Synagrops microlepis; Synchiropus phaeton; Alepocephalus rostratus; Nezumia sclerorhynchus* |
| 30 | Hake | *Merluccius senegalensis; Merluccius polli* |

## Table S2. Diet composition for the base model

|  | **Prey \ predator** | **1** | **2** | **3** | **4** | **5** | **6** | **7** | **8** | **9** | **10** | **11** | **12** | **13** | **14** | **15** |
| --- | --- | --- | --- | --- | --- | --- | --- | --- | --- | --- | --- | --- | --- | --- | --- | --- |
| 1 | Marine mammals | 0 | 0 | 0 | 0 | 0 | 0 | 0 | 0 | 0 | 0 | 0 | 0 | 0 | 0 | 0 |
| 2 | Coastal birds | 0 | 0 | 0 | 0 | 0 | 0 | 0 | 0 | 0 | 0 | 0 | 0 | 0 | 0 | 0 |
| 3 | Meagre ad | 0 | 0 | 0 | 0 | 0 | 0 | 0 | 0 | 0 | 0 | 00.10 | 0 | 0 | 0 | 0 |
| 4 | Meagre juv | 0 | 0.001 | 0 | 0 | 0 | 0 | 0 | 0 | 0 | 0 | 0 | 0 | 0 | 0.003 | 0 |
| 5 | Mullets | 4.81 | 2 | 7.69 | 8.33 | 0 | 0 | 0 | 0 | 0 | 0 | 5 | 0 | 0 | 0.096 | 0 |
| 6 | Pelagic L | 0 | 0.025 | 0 | 0 | 0 | 1.199 | 0 | 0 | 0 | 0 | 0 | 0 | 0 | 0.477 | 0 |
| 7 | Mackerel | 4.81 | 0 | 0 | 0 | 0 | 0.5 | 0 | 0 | 0 | 0 | 0 | 0 | 0 | 0 | 0 |
| 8 | Sardine | 4 | 0.654 | 0 | 0 | 0 | 18 | 3.0 | 0 | 0 | 0.10 | 2.4 | 0 | 0 | 4.077 | 0 |
| 9 | Sardinelles | 12 | 18 | 15.39 | 8.33 | 0 | 49 | 8.9 | 0 | 0 | 1 | 15.2 | 2.0 | 0 | 7.075 | 1.25 |
| 10 | Horse mackerels | 0 | 0 | 0 | 0 | 0 | 7 | 1.98 | 0 | 0 | 0.21 | 4.5 | 1.0 | 0 | 1.075 | 1.25 |
| 11 | Coastal selacians | 0 | 0 | 0 | 0 | 0 | 0 | 0 | 0 | 0 | 0 | 0.18 | 0 | 0 | 0 | 0 |
| 12 | Coastal M | 4.81 | 3.5 | 15.00 | 8.33 | 0 | 0.12 | 0.17 | 0 | 0 | 0 | 1.5 | 0.82 | 0 | 3.824 | 0 |
| 13 | Coastal S | 4.81 | 3.4 | 2.85 | 0 | 0 | 3 | 1 | 0 | 0 | 1 | 5 | 6.1 | 0 | 2.069 | 1.25 |
| 14 | Croakers ad | 0 | 0.5 | 3.85 | 0 | 0 | 0 | 0 | 0 | 0 | 0 | 0.10 | 0 | 0 | 1.425 | 0 |
| 15 | Croakers juv | 0 | 0.2 | 0.10 | 8.33 | 0 | 0 | 0 | 0 | 0 | 0 | 0.03 | 0 | 0 | 0 | 0 |
| 16 | Seabreams ad | 0 | 0 | 16.70 | 0 | 0 | 0.48 | 0 | 0 | 0 | 0 | 3.0 | 0 | 0 | 1.075 | 0 |
| 17 | Seabreams juv. | 0 | 0.2 | 0.50 | 8.33 | 0 | 0 | 0 | 0 | 0 | 0 | 0.06 | 0.1 | 0 | 0 | 0 |
| 18 | Catfish ad | 0 | 0 | 0 | 0 | 0 | 0 | 0 | 0 | 0 | 0 | 0 | 0.5 | 0 | 0 | 0 |
| 19 | Catfish juv | 0 | 0.03 | 0 | 8.33 | 0 | 0 | 0 | 0 | 0 | 0 | 0 | 0.0 | 0 | 0 | 0 |
| 20 | Shelf selacians | 0 | 0 | 0 | 0 | 0 | 0 | 0 | 0 | 0 | 0 | 0 | 0 | 0 | 0 | 0 |
| 21 | Shelf L | 0 | 0.01 | 0 | 0 | 0 | 0 | 0 | 0 | 0 | 0 | 0 | 0 | 0 | 0 | 0 |
| 22 | Shelf M | 4.81 | 0 | 7.69 | 8.33 | 0 | 0.24 | 0 | 0 | 0 | 0 | 1 | 0 | 0 | 2.417 | 0 |
| 23 | Groupers ad | 0 | 0 | 0 | 0 | 0 | 0 | 0 | 0 | 0 | 0 | 0.10 | 0.2 | 0 | 0.096 | 0 |
| 24 | Grouper juv | 0 | 0.0005 | 0 | 0 | 0 | 0 | 0 | 0 | 0 | 0 | 0.001 | 0 | 0 | 0 | 0 |
| 25 | Sparids ad | 0 | 1 | 3.30 | 0 | 0 | 1.319 | 0 | 0 | 0 | 0 | 0.50 | 0.4 | 0 | 1.171 | 0 |
| 26 | Sparids juv | 0 | 0.0005 | 0 | 0 | 0 | 0 | 0 | 0 | 0 | 0 | 0.09 | 0.05 | 0 | 0 | 0 |
| 27 | Scianids | 0 | 0.307 | 0 | 0 | 0 | 0.012 | 0 | 0 | 0 | 0 | 0 | 0 | 0 | 0.24 | 0 |
| 28 | Shelf soles | 4.81 | 2.4 | 3.85 | 8.33 | 0 | 0 | 0 | 0 | 0 | 0 | 0.10 | 0.10 | 0 | 1.306 | 0 |
| 29 | Shelf S | 4.84 | 2.917 | 0 | 0 | 0 | 6.2 | 0.25 | 0 | 0 | 5 | 9.5 | 3.6 | 0 | 1.120 | 0 |
| 30 | Octopus vulgaris | 0 | 0 | 0 | 0 | 0 | 0 | 0 | 0 | 0 | 0 | 0.45 | 0 | 0 | 0 | 0 |
| 31 | Cephalopods | 30.33 | 0.0001 | 7.69 | 4.167 | 0 | 0.7 | 2.0 | 0 | 0 | 0.20 | 3 | 0 | 0 | 1.438 | 0 |
| 32 | BA L crustaceans | 1.33 | 20 | 3.85 | 2.08 | 0 | 0 | 0.15 | 0 | 0 | 1.6 | 15 | 6 | 17 | 17.619 | 15.01 |
| 33 | BA molluscs | 0 | 20 | 0 | 0 | 6.44 | 0 | 0 | 0 | 0 | 0 | 2.1 | 7.60 | 1 | 0 | 0 |
| 34 | BA worms | 0 | 16 | 0 | 0 | 0 | 0 | 0.005 | 0 | 0 | 0 | 1.5 | 10.9 | 7 | 0 | 10.00 |
| 35 | BA crustaceans | 0 | 0.055 | 0 | 0 | 0 | 0 | 0.024 | 0 | 0 | 0.003 | 1.99 | 1.85 | 9 | 0 | 10.00 |
| 36 | BA other inverts | 0 | 0.1 | 0 | 0 | 0 | 0 | 0 | 0 | 0 | 0.014 | 0.09 | 2.00 | 0 |  |  |
| 37 | BA meiobenthos | 0 | 0.056 | 0 | 0 | 0 | 0 | 0 | 0 | 0 | 0 | 0 | 0 | 7.5 | 0 | 0 |
| 38 | shelf L crustaceans | 1.33 | 6.2 | 3.85 | 2.08 | 0 | 1 | 0.50 | 0 | 0 | 6 | 15.8 | 6.23 | 3 | 52.86 | 15.01 |
| 39 | shelf molluscs | 0 |  | 0 | 0 | 2.76 | 0.3 | 0 | 0 | 0 | 0 | 3.93 | 6.4 | 1 | 0 |  |
| 40 | shelf worms | 0 | 0 | 0 | 0 | 0 | 1.11 | 0.05 | 0 | 0 | 4.8 | 3.52 | 5.0 | 17.2 | 0 | 10.01 |
| 41 | shelf crustaceans | 0 | 0 | 0 | 0 | 0 | 0.8 | 0.24 | 0 | 0 | 0.02 | 4 | 2.09 | 12.77 | 0 | 10.01 |
| 42 | shelf other inverts | 0 | 0.035 | 0 | 0 | 0 | 0 | 0 | 0 | 0 | 0.05 | 0.09 | 4.75 | 0 | 0 | 0 |
| 43 | shelf meiobenthos | 0 | 0 | 0 | 0 | 0 | 0 | 0 | 0 | 0 | 0 | 0 | 0 | 7.5 | 0 | 0 |
| 44 | mesozooplankton | 0 | 0 | 0 | 5 | 0 | 0 | 55 | 90 | 66.76 | 35.5 | 0.07 | 2.4 | 7.33 | 0 | 10.2 |
| 45 | macrozooplankton | 0 | 0 | 3.85 | 7.5 | 0 | 7 | 17 | 0 | 0 | 15 | 0 | 1 | 1.18 | 0 | 3 |
| 46 | BA mesozoopl. | 0 | 0 | 0 | 5 | 0 | 0 | 2.4 | 0 | 11.1 | 6.50 | 0.07 | 2.7 | 7.33 | 0 | 10 |
| 47 | BA macrozoopl. | 0 | 0 | 3.85 | 7.5 | 0 | 0 | 2.13 | 0 | 0 | 15 | 0 | 4.68 | 1.18 | 0 | 3 |
| 48 | BA phytoplankton | 0 | 0 | 0 | 0 | 0 | 0 | 0 | 0 | 5.6 | 0 | 0 | 6.25 | 0 | 0 | 0 |
| 49 | phytoplankton | 0 | 0 | 0 | 0 | 0 | 0 | 0 | 10 | 16.7 | 0 | 0 | 1.56 | 0 | 0 | 0 |
| 50 | algae and eelgrass | 0 | 0 | 0 | 0 | 82.9 | 0 | 0 | 0 | 0 | 0 | 0 | 10.0 | 0 | 0 | 0 |
| 51 | Detritus | 0 | 0.422 | 0 | 0 | 7.9 | 0 | 5 | 0 | 0 | 1.956 | 0 | 3.71 | 0 | 0.523 | 0 |
| 52 | Import | 17.33 | 0.02 | 0 | 0 | 0 | 2.06 | 0.2 | 0 | 0 | 6.03 | 0 | 0 | 0 | 0 | 0 |

|  | **16** | **17** | **18** | **19** | **20** | **21** | **22** | **23** | **24** | **25** | **26** | **27** | **28** | **29** | **30** | **31** | **32** | **33** | **34** |
| --- | --- | --- | --- | --- | --- | --- | --- | --- | --- | --- | --- | --- | --- | --- | --- | --- | --- | --- | --- |
| 1 | 0 | 0 | 0 | 0 | 0 | 0 | 0 | 0 | 0 | 0 | 0 | 0 | 0 | 0 | 0 | 0 | 0 | 0 | 0 |
| 2 | 0 | 0 | 0 | 0 | 0 | 0 | 0 | 0 | 0 | 0 | 0 | 0 | 0 | 0 | 0 | 0 | 0 | 0 | 0 |
| 3 | 0 | 0 | 0 | 0 | 0 | 0 | 0 | 0 | 0 | 0 | 0 | 0 | 0 | 0 | 0 | 0 | 0 | 0 | 0 |
| 4 | 0 | 0 | 0 | 0 | 0 | 0 | 0 | 0 | 0 | 0 | 0 | 0 | 0 | 0 | 0 | 0 | 0 | 0 | 0 |
| 5 | 0 | 0 | 0 | 0 | 0.17 | 0 | 0 | 0 | 0 | 0 | 0 | 0 | 0 | 0 | 0 | 0 | 0 | 0 | 0 |
| 6 | 0 | 0 | 0 | 0 | 1.00 | 0 | 0.40 | 3.62 | 0 | 0 | 0 | 0 | 0 | 0 | 0 | 0 | 0 | 0 | 0 |
| 7 | 0 | 0 | 0 | 0 | 4.08 | 0 | 0.20 | 0 | 0 | 0 | 0 | 0 | 0 | 0 | 0 | 1.50 | 0 | 0 | 0 |
| 8 | 0 | 0 | 0 | 0 | 0.09 | 7.78 | 2.40 | 1.86 | 0 | 0 | 0 | 0 | 0 | 0 | 0 | 5.0 | 0 | 0 | 0 |
| 9 | 0 | 0.5 | 0 | 0 | 0.09 | 9.00 | 4.21 | 1.86 | 3.82 | 1.39 | 0 | 0 | 0 | 0 | 1 | 15.70 | 0 | 0 | 0 |
| 10 | 0 | 0 | 3.3 | 0 | 3.4 | 10.50 | 0.30 | 1.10 | 3.82 | 0 | 0 | 1.33 | 0 | 0 | 1 | 8.6 | 0 | 0 | 0 |
| 11 | 0 | 0 | 0 | 0 | 0 | 0 | 0 | 0 | 0 | 0 | 0 | 0 | 0 | 0 | 0 | 0 | 0 | 0 | 0 |
| 12 | 0.2 | 0 | 1 | 0 | 2.02 | 0.38 | 0.20 | 3.07 | 0 | 0.69 | 0 | 1.56 | 0.95 | 0 | 0 | 0 | 0 | 0 | 0 |
| 13 | 4.7 | 0.1 | 7 | 0 | 0.17 | 1.45 | 4.38 | 13.3 | 3.82 | 1.26 | 0 | 1.78 | 0.95 | 0.10 | 0 | 1.7 | 0 | 0 | 0 |
| 14 | 0 | 0 | 0.4 | 0 | 0.17 | 0.14 | 0 | 0 | 0 | 0 | 0 | 0 | 0 | 0 | 0 | 0 | 0 | 0 | 0 |
| 15 | 0 | 0.1 | 0 | 0 | 0 | 0 | 0 | 0 | 3.82 | 0 | 0 | 0 | 0 | 0 | 0 | 0 | 0 | 0 | 0 |
| 16 | 0 | 0 | 10 | 0 | 3.57 | 2.00 | 0.50 | 1.38 | 0 | 0 | 0 | 0.78 | 0 | 0 | 0 | 0 | 0 | 0 | 0 |
| 17 | 0 | 0 | 0.04 | 0 | 0 | 0 | 0 | 0 | 3.82 | 0.02 | 0 | 0 | 0 | 0 | 0 | 0 | 0 | 0 | 0 |
| 18 | 0 | 0 | 0 | 0 | 0 | 0 | 0 | 0 | 0 | 0 | 0 | 0 | 0 | 0 | 0 | 0 | 0 | 0 | 0 |
| 19 | 0 | 0 | 0 | 0 | 0 | 0 | 0 | 0 | 3.82 | 0 | 0 | 0 | 0 | 0 | 0 | 0 | 0 | 0 | 0 |
| 20 | 0 | 0 | 0 | 0 | 0.83 | 2.00 | 0 | 0 | 0 | 0 | 0 | 0 | 0 | 0 | 0 | 0 | 0 | 0 | 0 |
| 21 | 0 | 0 | 0 | 0 | 2.5 | 0 | 0 | 0 | 0 | 0 | 0 | 0 | 0 | 0 | 0 | 0 | 0 | 0 | 0 |
| 22 | 0 | 0 | 4 | 0 | 5.55 | 20.0 | 1.50 | 2.20 | 0 | 0 | 0 | 2.68 | 0 | 0 | 0 | 0.5 | 0 | 0 | 0 |
| 23 | 0 | 0 | 0 | 0 | 1.2 | 0.14 | 0 | 0 | 0 | 0 | 0 | 0.67 | 0 | 0 | 0 | 0 | 0 | 0 | 0 |
| 24 | 0 | 0 | 0 | 0 | 0 | 0.005 | 0 | 0 | 0 | 0 | 0 | 0 | 0 | 0 | 0 | 0 | 0 | 0 | 0 |
| 25 | 0 | 0 | 1.6 | 0 | 3.23 | 3.00 | 0 | 1.10 | 0 | 0 | 0 | 0.67 | 1.89 | 0 | 0 | 2 | 0 | 0 | 0 |
| 26 | 0 | 0 | 0.1 | 0 | 0 | 0.10 | 0 | 0 | 3.82 | 0 | 0 | 0.1 | 0 | 0 | 0 | 0.01 | 0 | 0 | 0 |
| 27 | 0 | 0 | 0.3 | 0 | 1.2 | 1.53 | 0 | 0.2 | 0 | 0 | 0 | 0 | 0 | 0 | 0 | 0 | 0 | 0 | 0 |
| 28 | 0.5 | 0 | 1 | 0 | 1.2 | 1.74 | 0.20 | 0.48 | 3.82 | 0 | 0 | 0 | 1.89 | 0 | 0 | 0 | 0 | 0 | 0 |
| 29 | 5.0 | 1 | 4.7 | 0 | 9.87 | 27.0 | 3.00 | 3.70 | 3.82 | 1.49 | 0 | 2.9 | 1.89 | 0.10 | 0 | 5.0 | 0 | 0 | 0 |
| 30 | 0.6 | 0.07 | 0 | 0 | 0.03 | 0 | 0 | 7.19 | 0 | 4.07 | 0 | 0 | 0 | 0 | 0 | 4 | 0 | 0 | 0 |
| 31 | 0.2 | 0.07 | 0 | 0 | 15.3 | 1.27 | 0 | 1.25 | 0 | 4.07 | 4.54 | 0 | 0 | 0.15 | 0.5 | 1.00 | 0 | 0 | 0 |
| 32 | 6 | 10 | 23 | 0 | 2.31 | 0 | 9.5 | 134 | 16.7 | 1.76 | 0 | 12.5 | 9 | 3 | 10 | 9 | 5 | 0 | 0 |
| 33 | 10 | 15 | 3 | 20 | 0 | 0 | 2.8 | 0.03 | 16.15 | 1.81 | 4.54 | 0 | 1.3 | 1.9 | 2 | 0 | 9 | 5 | 0 |
| 34 | 17.7 | 15 | 3 | 15 | 0.37 | 0 | 2 | 0 | 0 | 5.5 | 4.54 | 4.0 | 1 | 5.5 | 2 | 1 | 9 | 0 | 0 |
| 35 | 1.0 | 13 | 0 | 15 | 0.04 | 0 | 0.50 | 0 | 0 | 0.15 | 4.54 | 0.6 | 2.49 | 0.20 | 0 | 0.10 | 1.5 | 0 | 0 |
| 36 | 0.5 | 0.002 | 0 | 0 | 0 | 0 | 0.10 | 0.41 | 0 | 1.5 | 4.54 | 3.21 | 0 | 0.10 | 0 | 0 | 1 | 0 | 0 |
| 37 | 0.02 | 0 | 0 | 0 | 0.37 | 0 | 0 | 0 | 0 | 4.69 | 4.54 | 1.56 | 0.39 | 3.20 | 0 | 0 | 14.5 | 0 | 3 |
| 38 | 5.0 | 7.7 | 5 | 0 | 16.3 | 5.54 | 5.00 | 42 | 16.65 | 5.28 | 0 | 37.49 | 29.74 | 1.5 | 15 | 10.00 | 0 | 0 | 0 |
| 39 | 25.0 | 13.8 | 10 | 20 | 0 | 0 | 10.1 | 0.08 | 16.15 | 5.44 | 4.54 | 0 | 13.19 | 13 | 51.5 | 0 | 0 | 0 | 0 |
| 40 | 4.9 | 10 | 14 | 15 | 1.12 | 0 | 22.8 | 0 | 0 | 2.36 | 4.54 | 9.37 | 14.95 | 29.2 | 17 | 29.5 | 0 | 0 | 0 |
| 41 | 6.2 | 13.7 | 0 | 15 | 0.13 | 0.20 | 4.56 | 0 | 0 | 0.44 | 4.54 | 4.69 | 12.24 | 9 | 0 | 0.40 | 0 | 0 | 0 |
| 42 | 7.1 | 0.002 | 0 | 0 | 0 | 0.004 | 6.56 | 1.23 | 0 | 18.4 | 4.54 | 9.37 | 2.38 | 4 | 0 | 0 | 0 | 0 | 0 |
| 43 | 0.02 | 0 | 0 | 0 | 1.12 | 0 | 0 | 0 | 0 | 14.07 | 4.54 | 4.69 | 1.16 | 11.81 | 0 | 0 | 0 | 0 | 0 |
| 44 | 0 | 0 | 0 | 0 | 0 | 0.02 | 10.8 | 0 | 0 | 0 | 0 | 0 | 2 | 14.5 | 0 | 0 | 0 | 0 | 0 |
| 45 | 0 | 0 | 3.3 | 0 | 1.12 | 0.41 | 1 | 0 | 0 | 0 | 0 | 0 | 2 | 2 | 0 | 4.00 | 0 | 0 | 0 |
| 46 | 0 | 0 | 0 | 0 | 0 | 0 | 2.47 | 0 | 0 | 0 | 0 | 0 | 0 | 0.40 | 0 | 0 | 0 | 0 | 0 |
| 47 | 0 | 0 | 0 | 0 | 0.37 | 0 | 1.46 | 0 | 0 | 0 | 0 | 0 | 0.53 | 0.2 | 0 | 1.00 | 0 | 0 | 0 |
| 48 | 0 | 0 | 0 | 0 | 0 | 0 | 0 | 0 | 0 | 0 | 0 | 0 | 0 | 0 | 0 | 0 | 0 | 10 | 0 |
| 49 | 0 | 0 | 0 | 0 | 0 | 0 | 0 | 0 | 0 | 0 | 0 | 0 | 0 | 0 | 0 | 0 | 0 | 0 | 0 |
| 50 | 5.2 | 0 | 0 | 0 | 0 | 0 | 0 | 0 | 0 | 25.57 | 50 | 0 | 0 | 0 | 0 | 0 | 20 | 0 | 0 |
| 51 | 0 | 0 | 5 | 0 | 0 | 0 | 3.01 | 0 | 0 | 0 | 0 | 0 | 0 | 0 | 0 | 0 | 40 | 85 | 97 |
| 52 | 0 | 0 | 0 | 0 | 21.1 | 5.8 | 0 | 0 | 0 | 0 | 0 | 0 | 0 | 0.12 | 0 | 0 | 0 | 0 | 0 |

|  | **35** | **36** | **37** | **38** | **39** | **40** | **41** | **42** | **43** | **44** | **45** | **46** | **47** |
| --- | --- | --- | --- | --- | --- | --- | --- | --- | --- | --- | --- | --- | --- |
| 1 | 0 | 0 | 0 | 0 | 0 | 0 | 0 | 0 | 0 | 0 | 0 | 0 | 0 |
| 2 | 0 | 0 | 0 | 0 | 0 | 0 | 0 | 0 | 0 | 0 | 0 | 0 | 0 |
| 3 | 0 | 0 | 0 | 0 | 0 | 0 | 0 | 0 | 0 | 0 | 0 | 0 | 0 |
| 4 | 0 | 0 | 0 | 0 | 0 | 0 | 0 | 0 | 0 | 0 | 0 | 0 | 0 |
| 5 | 0 | 0 | 0 | 0 | 0 | 0 | 0 | 0 | 0 | 0 | 0 | 0 | 0 |
| 6 | 0 | 0 | 0 | 0 | 0 | 0 | 0 | 0 | 0 | 0 | 0 | 0 | 0 |
| 7 | 0 | 0 | 0 | 0 | 0 | 0 | 0 | 0 | 0 | 0 | 0 | 0 | 0 |
| 8 | 0 | 0 | 0 | 0 | 0 | 0 | 0 | 0 | 0 | 0 | 0 | 0 | 0 |
| 9 | 0 | 0 | 0 | 0 | 0 | 0 | 0 | 0 | 0 | 0 | 0 | 0 | 0 |
| 10 | 0 | 0 | 0 | 0 | 0 | 0 | 0 | 0 | 0 | 0 | 0 | 0 | 0 |
| 11 | 0 | 0 | 0 | 0 | 0 | 0 | 0 | 0 | 0 | 0 | 0 | 0 | 0 |
| 12 | 0 | 0 | 0 | 0 | 0 | 0 | 0 | 0 | 0 | 0 | 0 | 0 | 0 |
| 13 | 0 | 0 | 0 | 0 | 0 | 0 | 0 | 0 | 0 | 0 | 0 | 0 | 0 |
| 14 | 0 | 0 | 0 | 0 | 0 | 0 | 0 | 0 | 0 | 0 | 0 | 0 | 0 |
| 15 | 0 | 0 | 0 | 0 | 0 | 0 | 0 | 0 | 0 | 0 | 0 | 0 | 0 |
| 16 | 0 | 0 | 0 | 0 | 0 | 0 | 0 | 0 | 0 | 0 | 0 | 0 | 0 |
| 17 | 0 | 0 | 0 | 0 | 0 | 0 | 0 | 0 | 0 | 0 | 0 | 0 | 0 |
| 18 | 0 | 0 | 0 | 0 | 0 | 0 | 0 | 0 | 0 | 0 | 0 | 0 | 0 |
| 19 | 0 | 0 | 0 | 0 | 0 | 0 | 0 | 0 | 0 | 0 | 0 | 0 | 0 |
| 20 | 0 | 0 | 0 | 0 | 0 | 0 | 0 | 0 | 0 | 0 | 0 | 0 | 0 |
| 21 | 0 | 0 | 0 | 0 | 0 | 0 | 0 | 0 | 0 | 0 | 0 | 0 | 0 |
| 22 | 0 | 0 | 0 | 0 | 0 | 0 | 0 | 0 | 0 | 0 | 0 | 0 | 0 |
| 23 | 0 | 0 | 0 | 0 | 0 | 0 | 0 | 0 | 0 | 0 | 0 | 0 | 0 |
| 24 | 0 | 0 | 0 | 0 | 0 | 0 | 0 | 0 | 0 | 0 | 0 | 0 | 0 |
| 25 | 0 | 0 | 0 | 0 | 0 | 0 | 0 | 0 | 0 | 0 | 0 | 0 | 0 |
| 26 | 0 | 0 | 0 | 0 | 0 | 0 | 0 | 0 | 0 | 0 | 0 | 0 | 0 |
| 27 | 0 | 0 | 0 | 0 | 0 | 0 | 0 | 0 | 0 | 0 | 0 | 0 | 0 |
| 28 | 0 | 0 | 0 | 0 | 0 | 0 | 0 | 0 | 0 | 0 | 0 | 0 | 0 |
| 29 | 0 | 0 | 0 | 0 | 0 | 0 | 0 | 0 | 0 | 0 | 0 | 0 | 0 |
| 30 | 0 | 0 | 0 | 0 | 0 | 0 | 0 | 0 | 0 | 0 | 0 | 0 | 0 |
| 31 | 0 | 0 | 0 | 0 | 0 | 0 | 0 | 0 | 0 | 0 | 0 | 0 | 0 |
| 32 | 0 | 0 | 0 | 0 | 0 | 0 | 0 | 0 | 0 | 0 | 0 | 0 | 0 |
| 33 | 0 | 0 | 0 | 0 | 0 | 0 | 0 | 0 | 0 | 0 | 0 | 0 | 0 |
| 34 | 0 | 18 | 0 | 0 | 0 | 0 | 0 | 0 | 0 | 0 | 0 | 0 | 0 |
| 35 | 0 | 2 | 0 | 0 | 0 | 0 | 0 | 0 | 0 | 0 | 0 | 0 | 0 |
| 36 | 0 | 0 | 0 | 0 | 0 | 0 | 0 | 0 | 0 | 0 | 0 | 0 | 0 |
| 37 | 0 | 0 | 0 | 0 | 0 | 0 | 0 | 0 | 0 | 0 | 0 | 0 | 0 |
| 38 | 0 | 0 | 0 | 2 | 0 | 0 | 0 | 0 | 0 | 0 | 0 | 0 | 0 |
| 39 | 0 | 0 | 0 | 5 | 0 | 0 | 0 | 2 | 0 | 0 | 0 | 0 | 0 |
| 40 | 0 | 0 | 0 | 15 | 0 | 0 | 0 | 4 | 0 | 0 | 0 | 0 | 0 |
| 41 | 0 | 0 | 0 | 8 | 0 | 0 | 0 | 2 | 0 | 0 | 0 | 0 | 0 |
| 42 | 0 | 0 | 0 | 5 | 0 | 0 | 0 | 0 | 0 | 0 | 0 | 0 | 0 |
| 43 | 0 | 0 | 0 | 15 | 0 | 0.1 | 0 | 2 | 0 | 0 | 0 | 0 | 0 |
| 44 | 0 | 0 | 0 | 0 | 0 | 0 | 10 | 0 | 0 | 0 | 36 | 0 | 0 |
| 45 | 0 | 0 | 0 | 0 | 0 | 0 | 0 | 0 | 0 | 0 | 3 | 0 | 0 |
| 46 | 10 | 0 | 0 | 0 | 0 | 0 | 0 | 0 | 0 | 0 | 0 | 0 | 32 |
| 47 | 0 | 0 | 0 | 0 | 0 | 0 | 0 | 0 | 0 | 0 | 0 | 0 | 6 |
| 48 | 0 | 0 | 0 | 0 | 0 | 0 | 0 | 0 | 0 | 0 | 0 | 90 | 41 |
| 49 | 0 | 0 | 0 | 0 | 40 | 0 | 0 | 0 | 0 | 90 | 40 | 0 | 0 |
| 50 | 0 | 0 | 0 | 0 | 0 | 0 | 0 | 0 | 0 | 0 | 0 | 0 | 0 |
| 51 | 90 | 80 | 100 | 50 | 60 | 100 | 90 | 90 | 100 | 10 | 21 | 10 | 21 |
| 52 | 0 | 0 | 0 | 0 | 0 | 0 | 0 | 0 | 0 | 0 | 0 | 0 | 0 |

## Table S3. Effort used for each fleet

| **Year** | **Artisanal** | **Industrial demersal** | **Industrial pelagic** |
| --- | --- | --- | --- |
|  | **N boats ^a^** | **N boats ^a^** | **mean GRT ^b^** |
| 1991 | 742 | 160 | 2703 |
| 1992 | 681 | 190 | 3223 |
| 1993 | 1217 | 219 | 3630 |
| 1994 | 1540 | 251 | 4501 |
| 1995 | 1986 | 317 | 4730 |
| 1996 | 2587 | 318 | 4407 |
| 1997 | 2728 | 327 | 4200 |
| 1998 | 3142 | 312 | 4523 |
| 1999 | 2640 | 289 | 4704 |
| 2000 | 2750 | 267 | 5087 |
| 2001 | 2850 | 335 | 5002 |
| 2002 | 3700 | 357 | 4882 |
| 2003 | 3800 | 334 | 4980 |
| 2004 | 3950 | 328 | 4898 |
| 2005 | 3950 | 302 | 5003 |
| 2006 | *3950* | *302* | 5148 |
| ^a^. [[6](#_ENREF_6)]  ^b^. [[16](#_ENREF_16)] | | | |

## Table S4. Composition of the marine mammal group and parameters values for biomass, and P/B and Q/B ratios

| **English name** | **Latin name** | **Biomass**  **t/km^2^** |  | **P/B**  **/year** |  | **Q/B**  **/year** ^e^ |
| --- | --- | --- | --- | --- | --- | --- |
| Killer whale | *Orcinus orca* | 0.0026 | ^a^ | **0.02** | ^c^ | **7.58** |
| Monk seal | *Monachus monachus* | 0.0020 | ^b^ | 0.05 | ^d^ | 12.11 |
| Harbour porpoise | *Phocoena phocoena* |  |  |  |  | 14.18 |
| Dolphins, pilot whales, and pigmy sperm whale | *Sousa teuszii, Grampus griseus, Tursiops truncatus, Stenella frontalis, Stenella attenuata, Stenella longirostris, Stenella coeuruleoalba, Stenella clymene, Delphinus delphis, Globicephala melas, Globicephala macrorhincus, Kogia breviceps* | 0.0001 | ^a^ | 0.036 | ^a^ | 9.92 |
| Beaked whales | *Mesoplodon densirostris, M. europaeus, Ziphius cavirostris* | 0.0001 | ^a^ | 0.036 | ^a^ | 9.92 |
| **Weighted average/sum** | | **0.0076** |  | **0.05** |  | **12.56** |
| ^a.^*.* [[17](#_ENREF_17)]  ^b^*.* [[18](#_ENREF_18)]  *^c^*. [[19](#_ENREF_19)]  ^d^*.* the P/B is assumed equal to that of dolphins (0.05)  ^e^. Calculated from ration (*R*) in % of body weight per day [[19](#_ENREF_19)]: *R=0.1*W^0.8^* where *W* is the body weight in kg taken from [[50](#_ENREF_50)] | | | | | | |

## Table S5. Composition of the coastal bird groups and parameters used in the model.

BW is body weight

| **Latin name** | **Common name** | **N pairs** |  | **N summer** | **N winter** | | **residence** | | **BW (kg)** | | **Biom. (t)** | **QB**  **/year** | **M /year** | |
| --- | --- | --- | --- | --- | --- | --- | --- | --- | --- | --- | --- | --- | --- | --- |
| **Breeders** |  |  |  |  |  |  |  |  |  |  |  |  |  |  |
| *Pelecanus onocrotalus* | White pelican | 3080 | ^a^ | 9240 |  |  | 12 | ^f^ | 10.50 | ^b^ | 97 | 46 |  |  |
| *Phoenicopterus ruber* | Greater flamingo | 12940 | ^a^ | 38820 |  |  | 6 | ^f^ | 3.00 | ^e^ | 58 | 56 |  |  |
| *Microcarbo africanus* | Long-tailed cormorant | 2460 | ^a^ | 7380 |  |  | 6 | ^f^ | 0.62 | ^e^ | 2 | 71 | 0.10 | v |
| *Phalacrocorax carbo lucidus* | Cormorant | 4260 | ^a^ | 12780 |  |  | 12 | ^f^ | 2.25 |  | 29 | 58 |  |  |
| *Platalea leucorodia* | Spoonbill | 1610 | ^a^ | 4830 |  |  | 12 | ^f^ | 1.36 | ^e^ | 7 | 63 | 0.19 | w |
| *Ardea cinera* | Grey heron | 2400 | ^a^ | 7200 |  |  | 12 | ^f^ | 0.90 | ^e^ | 6 | 67 | 0.36 | l |
| *Egretta garzetta/gularis* | Little egret/reef heron | 745 | ^a^ | 2235 |  |  | 12 | ^f^ | 0.50 | ^e^ | 1 | 73 |  |  |
| *Larus genei* | Slender-billed gull | 1610 | ^a^ | 4830 |  |  | 12 | ^f^ | 0.30 | ^e^ | 1 | 79 |  |  |
| *Chroicocephalus cirrocephalus* | Grey-headed gull | 15 | ^a^ | 45 |  |  | 12 | ^f^ |  |  | 0 |  |  |  |
| *Gelochiledon nilotica* | Gull-billed tern | 1180 | ^a^ | 3540 |  |  | 12 | ^f^ | 0.27 | ^x^ | 1 |  |  |  |
| *Hydroprogne caspia* | Caspian tern | 2575 | ^a^ | 7725 |  |  | 12 | ^f^ | 0.65 | ^e^ | 5 | 70 | 0.13 | l |
| *Thalasseus maximus* | Royal tern | 5630 | ^a^ | 16890 |  |  | 12 | ^f^ | 0.40 | ^e^ | 7 | 76 |  |  |
| *Sterna albifrons* | Little tern | 30 | ^a^ | 90 |  |  | 12 | ^f^ | 0.06 | ^e^ | 0 | 101 |  |  |
| *Sterna hirundo* | Common tern | 100 | ^a^ | 300 |  |  | 6 | ^f^ | 0.12 | ^e^ | 0 | 91 | 0.32 | l |
| *Onychoprion anaethetus* | Bridled tern | 440 | ^a^ | 1320 |  |  | 8 | ^b^ | 0.12 | ^u^ | 0 |  |  |  |
|  | **total breeders** |  |  |  |  |  |  |  |  |  | **215** | **54** | **0.22** |  |
| **Waders** |  |  |  |  |  |  |  |  |  |  |  |  |  |  |
| *Haematopus ostralegus* | Oystercatcher |  |  |  | 129 | ^r, s^ | 8 | ^b^ | 0.55 | ^e^ | 1 | 72 |  |  |
| *Charadrius hiaticula* | Ringed plover |  |  |  | 1805 | ^r, s^ | 8 | ^b^ | 0.05 | ^b^ | 2 | 104 |  |  |
| *Charadrius alexandrinus* | Kentish plover |  |  |  | 85 | ^r, s^ | 8 | ^b^ | 0.04 | ^b^ | 0 | 108 | 0.22 | l |
| *Pluvialis squatarola* | Grey plover |  |  |  | 457 | ^r, s^ | 8 | ^b^ | 0.19 | ^b^ | 1 | 85 |  |  |
| *Calidris canutus* | Knot |  |  |  | 26635 | ^r, s^ | 8 | ^b^ | 0.12 | ^b^ | 54 | 91 |  |  |
| *Calidris alba?* | Sanderling |  |  |  | 484 | ^r, s^ | 8 | ^b^ | 0.05 | ^b^ | 0 | 104 |  |  |
| *Calidris minuta* | Little stint |  |  |  | 700 | ^r, s^ | 8 | ^b^ | 0.02 | ^b^ | 0 | 117 |  |  |
| *Calidris ferruginea* | Curlew Sandpiper |  |  |  | 7770 | ^r, s^ | 8 | ^b^ | 0.05 | ^b^ | 7 | 103 |  |  |
| *Calidris alpina* | Dunlin |  |  |  | 28936 | ^r, s^ | 8 | ^b^ | 0.04 | ^b^ | 21 | 106 |  |  |
| *Limosa lapponica* | Bar-tailed godwit |  |  |  | 5756 | ^r, s^ | 8 | ^b^ | 0.03 | ^b^ | 2 | 114 |  |  |
| *Numenius phaeopus* | Whimbrel |  |  |  | 1048 | ^r, s^ | 8 | ^b^ | 0.41 | ^b^ | 7 | 75 |  |  |
| *Numenius arquata* | Curlew |  |  |  | 213 | ^r, s^ | 8 | ^b^ | 0.72 | ^b^ | 3 | 69 |  |  |
| *Tringa totanus* | Redshanks |  |  |  | 4765 | ^r, s^ | 8 | ^b^ | 0.11 | ^b^ | 9 | 92 | 0.29 | l |
| *Tringa nebularia* | Greenshank |  |  |  | 88 | ^r, s^ | 8 | ^b^ | 0.18 | ^b^ | 0 | 85 |  |  |
| *Arenaria interpres* | Turnstone |  |  |  | 408 | ^r, s^ | 8 | ^b^ | 0.10 | ^b^ | 1 | 93 |  |  |
|  | **total waders** |  |  |  |  |  |  |  |  |  | **109** | **93** | **0.46** |  |
|  | **coastal birds** |  |  |  |  |  |  |  |  |  | **324** | **67** | **0.30** |  |
| ^a^ Campredon 1987 (for year 1984-85) in [[23](#_ENREF_23)]. Npairs* 3 to estimate the whole population (Nsummer)  ^b^ [[23](#_ENREF_23)]  ^c^ [[51](#_ENREF_51)]; May 1988 shelf edge bordering BA; 20->1000m  ^d^ count on Feb 1997, [[24](#_ENREF_24)].  ^e^ http://www.oiseaux.net/oiseaux/  ^f^ based on [[52](#_ENREF_52)]; species that are present in both winter and summer are assumed to be present 12 months, averaging abundances  ^g^ assumed  ^h^ preparing to migrate in May so assumed to have spent winter in BA  ^i^ start arriving in Oct, mainly present Dec-Feb  ^j^ winters further south, migrate through from March to May  ^k^ [[53](#_ENREF_53)]  ^l^ from survival estimate from [[27](#_ENREF_27)]  ^m^ [[54](#_ENREF_54)]  ^n^ based on Leach's storm petrel minimum as weight is max weight from sept-nov in Mauritania [[55](#_ENREF_55)]  ^p^ [[56](#_ENREF_56)]  ^q^ [[57](#_ENREF_57)]  ^r^ [[24](#_ENREF_24)]  ^s^ original counts in Baie Arguin; multiplied by 25.5 to account for surface in the rest of the Banc Arguin  ^t^ present in the fall and in May  ^u^ assumed equal to common tern  ^v^ from the survival value for adults in [[26](#_ENREF_26)] for unspecified cormorants  ^w^ from the survival value for adults in [[28](#_ENREF_28)]  ^x^ average body weights of other sterns  ^y^ obtained from the equation Log(Ration)=-0.293+0.85*Log(BW) [[25](#_ENREF_25)] where BW is the body weight in kg. | | | | | | | | | | | | | | |

## Table S6. Parameters used to calculate benthos biomass and production by unit of biomass (P/B).

AFDW: Ash-free dry weight; DW: dry weight; WW: wet-weight; surf adj: adjusted for surface ratio;

|  |  | | **Banc d'Arguin** | | | | |  | **Biomass shelf** | | | | | | | |  |  |
| --- | --- | --- | --- | --- | --- | --- | --- | --- | --- | --- | --- | --- | --- | --- | --- | --- | --- | --- |
| **Group** | **AFDW/WM** |  | **AFDW g/m^2^** |  | **WW g/m^2^** | **surf adj** |  |  | **AFDW/g/m^2^** |  | **N 106/m^2^ m** | | **body weight (micro g)** | | **DW/WW** | **WW g/m^2^** | **P/B**  **/year** | |
| Crustaceans |  |  |  |  | 0.79 | 0.79 | ^v^ |  | 1.49 | ^j,k^ |  |  |  |  |  | 9.92 | 2.4 | ^q^ |
| Amphipoda | 0.15 | ^a^ | 0.0348 | ^b^ | 0.23 |  |  |  |  |  |  |  |  |  |  |  |  |  |
| Crustacea | 0.15 | ^a^ | 0.0022 | ^b^ | 0.01 |  |  |  |  |  |  |  |  |  |  |  |  |  |
| Isopoda | 0.15 | ^a^ | 0.0479 | ^b^ | 0.32 |  |  |  |  |  |  |  |  |  |  |  |  |  |
| Sipunculida | 0.15 | ^a^ | 0.0342 | ^b^ | 0.23 |  |  |  |  |  |  |  |  |  |  |  |  |  |
| Tanaidacea | 0.15 | ^a^ | 0.0007 | ^b^ | 0.00 |  |  |  |  |  |  |  |  |  |  |  |  |  |
| Molluscs |  |  |  |  | 132.55 | 94.00 |  |  | 4.84 | ^j^ |  |  |  |  |  | 32.36 | 1.5 |  |
| Gastropoda | 0.16 | ^c^ | 1.8521 | ^b^ | 11.36 | 11.36 | ^u^ |  |  |  |  |  |  |  |  |  | 1.5 |  |
| Bivalvia | 0.14 | ^c^ | 10.981 | ^b^ | 80.74 | 80.74 | ^u^ |  |  |  |  |  |  |  |  |  | 1.5 | ^r^ |
| Senilia senilis | 0.14 | ^c^ | 8.1000 | ^b^ | 40.44 | 1.90 | ^v^ |  |  |  |  |  |  |  |  |  | 0.02 |  |
| Worms |  |  |  |  | 28.00 | 28.00 | ^u^ |  |  |  |  |  |  |  |  | 39.22 | 3 | ^q^ |
| Nemertinea | 0.17 | ^c^ | 0.0132 | ^b^ | 0.08 | 0.08 |  |  |  |  |  |  |  |  |  |  |  |  |
| Oligocheta | 0.05 | ^c^ | 0.1044 | ^b^ | 1.90 | 1.90 |  |  | 0.96 | ^j,l^ |  |  |  |  |  | 0.05 |  |  |
| Planaria | 0.05 | ^f^ | 0.0019 | ^b^ | 0.03 | 0.03 |  |  |  |  |  |  |  |  |  |  |  |  |
| Polychaeta | 0.15 | ^c^ | 3.8972 | ^b^ | 25.98 | 25.98 |  |  | 5.88 | ^j^ |  |  |  |  |  | 39.17 |  |  |
| Other inverts |  |  |  |  | 0.55 | 0.55 | ^v^ |  |  |  |  |  |  |  |  | 21.25 |  |  |
| Coelenterata | 0.12 | ^c,d^ | 0.0019 | ^b^ | 0.02 |  |  |  |  |  |  |  |  |  |  |  |  |  |
| Echinodermata | 0.11 | ^c,e^ | 0.0581 | ^b^ | 0.52 |  |  |  |  |  |  |  |  |  |  |  |  |  |
| Holothuroidea | 0.077 | ^c^ |  |  |  |  |  |  |  |  |  |  |  |  |  |  |  |  |
| Ophiuroidea | 0.148 | ^c^ |  |  |  |  |  |  | 3.14 | ^j^ |  |  |  |  |  | 21.20 | 1.8 | ^q^ |
| Insecta | 0.16 | ^c^ | 0.0023 | ^b^ | 0.01 |  |  |  |  |  |  |  |  |  |  |  |  |  |
| Miscellaneous |  |  |  |  |  |  |  |  | 0.45 | ^s^ |  |  |  |  |  | 0.05 |  |  |
| Total macrobenthos | |  | 17.031 |  | 161.89 | 123.34 |  |  | 16.8 |  |  |  |  |  |  | 196.3 |  |  |
| Meiobenthos | |  |  |  | 9.07 | 6.91 |  |  |  |  |  |  |  |  |  | 11.0 | 9 | ^p^ |
| Meiobenthos |  |  |  |  | 9.07 | 6.91 | ^h^ |  |  |  |  |  |  |  |  |  |  |  |
| Nematodes |  |  |  |  |  |  |  |  |  |  | 2.56 | ^j^ | 0.453 DW | ^n^ | 0.25 | 4.6 |  |  |
| Copepods |  |  |  |  |  |  |  |  |  |  | 0.41 | ^j^ | 1.90 DW | ^o^ | 0.214 | 3.7 |  |  |
| Foraminiferans |  |  |  |  |  |  |  |  |  |  | 2.70 | ^j^ | 1 WW | ^p^ | na | 2.7 |  |  |
| Crustaceans L |  |  |  |  |  | 51.67 |  |  |  |  |  |  |  |  |  |  |  |  |
| *Uca tangeri* | 0.75 | ^c, g^ | 0.589 | ^i^ | 1.27 | 0.06 |  |  |  |  |  |  |  |  |  |  |  |  |
| Shrimp |  | ^t^ |  |  | 51.61 | 51.61 |  |  |  |  |  |  |  |  |  |  |  |  |
| ^a^ value used for crustaceans by [[41](#_ENREF_41)]  ^b^ Values for tidal flats from Table 3 in [[41](#_ENREF_41)]; the surface of tidal flats is assumed to be 500 km^2^ [[42](#_ENREF_42)]  ^c^ from [[58](#_ENREF_58)], molluscs conversion without shell  ^d^ using Actinaria as an example  ^e^ average of holothurians and Ophiuridae based on species list  ^f^ assumed equal to that of Oligocheta  ^g^ based on *Uca lactea*  ^h^ assumed that meiobenthos biomass was in the same proportion as the ratio of meiobentho/macrobenthos as on the shelf. By comparison, Wollf et al [[41](#_ENREF_41)] estimate that 3-10% of benthos is not retained in their sieve  ^i^  0.5-1 g AFDW/m^2^ [[41](#_ENREF_41)]  ^j^ [[40](#_ENREF_40)]  ^k^ Isopoda,Cumacea, Gammaridae, Tanaidacea  ^l^ assumed to be mainly Oligochaeta and that the same density of other inverts were present on the shelf as on tidal flats  ^m^ [[40](#_ENREF_40)]  ^n^ [[59](#_ENREF_59)]; St Lawrence River, Canada. coastal nematods are larger than deep-sea; 0.44 and 0.37 microg in Cretan Sea [[60](#_ENREF_60)]  ^o^ based on average of size range observed in figure 1 in [[61](#_ENREF_61)]  ^p^ assumed same value as in [[45](#_ENREF_45)]  ^q^ based on empirical equation [[44](#_ENREF_44)] which yielded similar results as that of [[43](#_ENREF_43)]  ^r^ assumed value  ^s^ assumed same density of other invertebrates as on tidal flats  t push net sampling with correction for under-sampling [[42](#_ENREF_42)]. Nevertheless, Penaeus sp are probably underestimated as well as individuals smaller than 10 mm. Assumed individual body weight of 0.5 g for *Hippolyte inermis* and 1 g for Palaemon spp. and Penaeus spp.  ^u^ Biomass for the entire Banc =Btidal (P+0.5(1-P)) where P is the proportion of the tidal flats in the Banc d'Arguin =0.05, and assuming that the biomass was half as abundant in subtidal habitats | | | | | | | | | | | | | | | | | | |

## Table S7. List of biomass and catch time series available and used in the Ecosim fitting process.

n/a: not available

|  |  | **Time series** | |
| --- | --- | --- | --- |
|  | **Group name** | **Biomass** | **Catch** |
| 1 | Marine mammals | n/a | n/a |
| 2 | Coastal birds | n/a | n/a |
| 3 | Meagre ad | n/a | available |
| 4 | Meagre juv | n/a | n/a |
| 5 | Mullets | n/a | available |
| 6 | Pelagic L | n/a | available |
| 7 | Mackerel | n/a | available |
| 8 | Sardine | not used | available |
| 9 | Sardinelles | available | available |
| 10 | Horse mackerels | not used | available |
| 11 | Coastal selacians | available | available |
| 12 | Coastal M | available | available |
| 13 | Coastal S | n/a | n/a |
| 14 | Croakers ad | available | available |
| 15 | Croakers juv |  | n/a |
| 16 | Seabreams ad | available | available |
| 17 | Seabreams juv. | n/a | n/a |
| 18 | Catfish ad | not used | available |
| 19 | Catfish juv | n/a | n/a |
| 20 | Shelf selacians | available | available |
| 21 | Shelf L | available | available |
| 22 | Shelf M | available | available |
| 23 | Groupers ad | available | available |
| 24 | Grouper juv | n/a | n/a |
| 25 | Sparids ad | available | available |
| 26 | Sparids juv | n/a | n/a |
| 27 | Scianids | available | available |
| 28 | Shelf soles | available | available |
| 29 | Shelf S | available | available |
| 30 | Octopus vulgaris | available | available |
| 31 | Cephalopods | available | available |
| 32 | BA L crustaceans | available | n/a |
| 33 | BA molluscs | n/a | n/a |
| 34 | BA worms | n/a | n/a |
| 35 | BA crustaceans | n/a | n/a |
| 36 | BA other inverts | n/a | n/a |
| 37 | BA meiobenthos | n/a | n/a |
| 38 | shelf L crustaceans. | n/a | available |
| 39 | shelf molluscs | n/a | available |
| 40 | shelf worms | n/a | n/a |
| 41 | shelf crustaceans | n/a | n/a |
| 42 | shelf other inverts | n/a | n/a |
| 43 | shelf meiobenthos | n/a | n/a |
| 44 | mesozooplankton | n/a | n/a |
| 45 | macrozooplankton | n/a | n/a |
| 46 | BA mesozoopl. | n/a | n/a |
| 47 | BA macrozoopl. | n/a | n/a |
| 48 | BA phytoplankton | n/a | n/a |
| 49 | phytoplankton | n/a | n/a |
| 50 | algae and eelgrass | n/a | n/a |
| 51 | Detritus | n/a | n/a |

## Table S8. Proportion of invertebrates from the Banc (pBAi) in diets imposed in each model (M30, Base and P30), and resulting biomass, ecotrophic efficiency (EE) from balancing the Ecopath model, and vulnerability values for each Ecosim model (M30, Base and P30) fitted to the time series.

Only vulnerability values different from the default value (2) are listed. Values in bold are biomass and EE that were calculated by Ecopath instead of being input.

|  |  |  | | | **Balanced Ecopath model** | | | | | | |  | | |
| --- | --- | --- | --- | --- | --- | --- | --- | --- | --- | --- | --- | --- | --- | --- |
|  |  | **pBAi (imposed)** | | | **M30** | | | **Base** | | **P30** | | **Vulnerability (Ecosim)** | | |
|  | **Group name** | **M30** | **Base** | **P30** | | **Biom** | **EE** | **Biom** | **EE** | **Biom** | **EE** | **M30** | **Base** | **P30** |
| 1 | Marine mammals | 0.5 | 0.5 | 0.5 | | 0.01 | 0 | 0.01 | 0 | 0.01 | 0 |  |  |  |
| 2 | Coastal birds | 1 | 1 | 1 | | 0.01 | 0 | 0.01 | 0 | 0.01 | 0 |  |  |  |
|  | **Meagre** |  |  |  | |  |  |  |  |  |  |  |  |  |
| 3 | Meagre ad | 0.33 | 0.5 | 0.75 | | 0.12 | 0.66 | 0.12 | 0.66 | 0.12 | 0.66 | 1.002 | 1.01 | 1.004 |
| 4 | Meagre juv | 0.33 | 0.5 | 1 | | 5E-05 | 0.97 | 5.3E-05 | 0.97 | 5E-05 | 0.97 |  |  |  |
| 5 | Mullets | 0.47 | 0.7 | 1 | | 0.42 | 0.8 | 0.42 | 0.80 | 0.42 | 0.8 | 1 | 1 | 1 |
| 6 | Pelagic L | 0 | 0 | 0 | | **3.42** | **0.9** | **3.42** | **0.90** | **3.89** | **0.8** | 1.713 | 7.205 | 4.473 |
| 7 | Mackerel | 0.1 | 0.1 | 0.1 | | 1.45 | 0.74 | 1.45 | 0.74 | 1.45 | 0.75 | 1 | 1.002 | 1.05 |
| 8 | Sardine | 0 | 0 | 0 | | 11.79 | 0.77 | 11.79 | 0.77 | 11.79 | 0.83 | 1 | 2 | 2 |
| 9 | Sardinelles | 0.25 | 0.25 | 0.25 | | 18 | 0.78 | 18 | 0.78 | 18 | 0.85 | 1 | 1 | 1 |
| 10 | Horse mackerels | 0.25 | 0.25 | 0.25 | | 10 | 0.84 | 10 | 0.84 | 10 | 0.87 | 1 | 2.144 | 2.002 |
| 11 | Coastal selacians | 0.33 | 0.5 | 0.75 | | 1.24 | 0.01 | 1.24 | 0.01 | 1.24 | 0.01 | 2.338 | 2.265 | 2.365 |
| 12 | Coastal M | 0.33 | 0.5 | 0.75 | | 0.83 | 0.86 | 0.83 | 0.86 | 0.83 | 0.86 | 1.12 | 1.11 | 1.179 |
| 13 | Coastal S | 0.33 | 0.5 | 0.75 | | 4.21 | 0.95 | **4.21** | **0.95** | **5.18** | **0.8** | 1.477 | >100 | >100 |
|  | **Croakers** |  |  |  | |  |  |  |  |  |  |  |  |  |
| 14 | Croakers ad | 0.17 | 0.25 | 0.375 | | 0.08 | 0.75 | 0.077 | 0.75 | 0.08 | 0.75 | 1.101 | 1 | 1 |
| 15 | Croakers juv | 0.33 | 0.5 | 1 | | 0.00 | 0.71 | 0.00352 | 0.71 | 0.00 | 0.71 |  |  |  |
|  | **Seabreams** |  |  |  | |  |  |  |  |  |  |  |  |  |
| 16 | Seabreams ad | 0.33 | 0.5 | 0.75 | | 1.69 | 0.88 | 1.69 | 0.88 | 1.69 | 0.90 | 1.574 | 1.838 | 2.598 |
| 17 | Seabreams juv. | 0.33 | 0.5 | 1 | | 0.01 | 0.88 | 0.0125 | 0.88 | 0.01 | 0.88 |  |  |  |
|  | **Catfish** |  |  |  | |  |  |  |  |  |  |  |  |  |
| 18 | Catfish ad | 0.33 | 0.5 | 0.75 | | 0.60 | 0.23 | 0.6 | 0.23 | 0.60 | 0.23 | 1 | 1 | 1.005 |
| 19 | Catfish juv | 0.33 | 0.5 | 1 | | 0.00 | 0.78 | 0.00166 | 0.78 | 0.00 | 0.78 |  |  |  |
| 20 | Shelf selacians | 0.25 | 0.25 | 0.25 | | 0.20 | 0.79 | 0.2 | 0.79 | 0.20 | 0.79 | 1 | 1 | 1 |
| 21 | Shelf L | 0 | 0 | 0 | | 0.36 | 0.47 | 0.36 | 0.47 | 0.36 | 0.47 | 1 | 1 | 1 |
| 22 | Shelf M | 0.25 | 0.25 | 0.25 | | 1.55 | 0.91 | 1.55 | 0.91 | 1.55 | 0.91 | 1 | 1 | 1.048 |
|  | **Groupers** |  |  |  | |  |  |  |  |  |  |  |  |  |
| 23 | Groupers ad | 0.25 | 0.25 | 0.25 | | 0.11 | 0.94 | 0.11 | 0.94 | 0.11 | 0.94 | 1.03 | 1.039 | 1.037 |
| 24 | Grouper juv | 0.33 | 0.5 | 1 | | 0.00 | 0.54 | 0.00037 | 0.54 | 0.00 | 0.54 |  |  |  |
|  | **Sparids** |  |  |  | |  |  |  |  |  |  |  |  |  |
| 25 | Sparids ad | 0.25 | 0.25 | 0.25 | | 1.29 | 0.87 | 1.29 | 0.87 | 1.29 | 0.93 | 1 | 1 | 1 |
| 26 | Sparids juv | 0.33 | 0.5 | 1 | | 0.011 | 0.94 | 0.01074 | 0.94 | 0.01 | 0.94 |  |  |  |
| 27 | **Scianids** | 0.25 | 0.25 | 0.25 | | 0.22 | 0.67 | 0.22 | 0.67 | 0.22 | 0.67 | >100 | >100 | >100 |
| 28 | Shelf soles | 0.25 | 0.25 | 0.25 | | 0.35 | 0.88 | 0.35 | 0.88 | 0.35 | 0.88 | 1 | 1 | 1 |
| 29 | Shelf S | 0.25 | 0.25 | 0.25 | | 6.19 | 0.95 | **6.19** | **0.95** | **7.61** | **0.8** | 21.02 | >100 | >100 |
| 30 | Octopus vulgaris | 0.2 | 0.2 | 0.2 | | 1.37 | 0.63 | 1.37 | 0.63 | 1.37 | 0.63 | 4.45 | 1.116 | 1.132 |
| 31 | Cephalopods | 0.2 | 0.2 | 0.2 | | 1 | 0.84 | 1 | 0.84 | 1 | 0.87 | 2 | 4.165 | 4.065 |
| 32 | BA L crustaceans | 1 | 1 | 1 | | 7.66 | 0.88 | 9.12 | 0.88 | 11.02 | 0.83 |  |  |  |
| 33 | BA molluscs | 1 | 1 | 1 | | 14.01 | 0.93 | 17.86 | 0.89 | 22.86 | 0.91 |  |  |  |
| 34 | BA worms | 1 | 1 | 1 | | 4.15 | 0.90 | 5.32 | 0.81 | 6.84 | 0.86 |  |  |  |
| 35 | BA crustaceans | 1 | 1 | 1 | | 1.14 | 0.87 | 1.14 | 0.98 | 2.09 | 0.98 |  |  |  |
| 36 | BA other inverts | 1 | 1 | 1 | | 0.57 | 0.93 | 0.57 | 0.87 | 0.95 | 0.90 |  |  |  |
| 37 | BA meiobenthos | 1 | 1 | 1 | | 2.09 | 0.77 | 2.09 | 0.93 | 2.66 | 0.93 |  |  |  |
| 38 | shelf L crustac. | 0 | 0 | 0 | | **8.91** | 0.83 | 8.10 | 0.73 | 8.10 | 0.74 | 2 | 1.966 | 2.061 |
| 39 | shelf molluscs | 0 | 0 | 0 | | 26.21 | 0.51 | 26.21 | 0.50 | 26.21 | 0.49 |  |  |  |
| 40 | shelf worms | 0 | 0 | 0 | | 31.77 | 0.43 | 31.77 | 0.41 | 31.77 | 0.43 |  |  |  |
| 41 | shelf crustaceans | 0 | 0 | 0 | | 8.04 | 0.83 | 8.04 | 0.80 | 9.04 | 0.78 |  |  |  |
| 42 | shelf other inverts | 0 | 0 | 0 | | 17.21 | 0.23 | 17.21 | 0.22 | 17.21 | 0.22 |  |  |  |
| 43 | shelf meiobenthos | 0 | 0 | 0 | | 8.91 | 0.27 | 8.91 | 0.25 | 8.91 | 0.26 |  |  |  |
| 44 | mesozooplankton | 0 | 0 | 0 | | 55.08 | 0.15 | 55.08 | 0.15 | 55.08 | 0.15 |  |  |  |
| 45 | macrozooplankton | 0 | 0 | 0 | | 3.41 | 0.73 | 3.41 | 0.73 | 3.41 | 0.75 |  |  |  |
| 46 | BA mesozoopl. | 1 | 1 | 1 | | 1.75 | 0.8 | 1.78 | 0.8 | 1.91 | 0.8 |  |  |  |
| 47 | BA macrozoopl. | 1 | 1 | 1 | | 2.46 | 0.8 | 2.50 | 0.8 | 2.59 | 0.8 |  |  |  |
| 48 | BA phytoplankton |  |  |  | | 6 | 0.38 | 6 | 0.40 | 6 | 0.44 |  |  |  |
| 49 | phytoplankton |  |  |  | | 68 | 0.85 | 68 | 0.85 | 68 | 0.85 |  |  |  |
| 50 | algae and eelgrass |  |  |  | | 549 | 0.01 | 549 | 0.01 | 549 | 0.01 |  |  |  |
| 51 | Detritus |  |  |  | | 560 | 0.45 | 560 | 0.46 | 560 | 0.48 |  |  |  |

## Table S9. Biomass, catch and fishing mortality (C/B) by functional groups based on EwE data (1991) and estimations (2006).

| **no** | **Group name** | **Biomass 1991** | **Biomass 2006** | **Catch 1991** | **Catch 2006** | **C/B 1991** | **C/B 2006** |
| --- | --- | --- | --- | --- | --- | --- | --- |
| 1 | Marine mammals | 0.009999 | 0.008541 |  |  |  |  |
| 2 | Coastal birds | 0.010001 | 0.010549 |  |  |  |  |
| 3 | Meagre ad | 0.12004 | 0.059078 | 0.014005 | 0.024851 | 0.12 | 0.42 |
| 4 | Meagre juv | 5.34E-05 | 3.35E-05 |  |  |  |  |
| 5 | Mullets | 0.4206 | 0.339271 | 0.103147 | 0.268065 | 0.25 | 0.79 |
| 6 | Pelagic L | 3.420516 | 1.627229 | 2.679404 | 1.333773 | 0.78 | 0.82 |
| 7 | Mackerel | 1.450124 | 1.656654 | 0.289025 | 0.341271 | 0.20 | 0.21 |
| 8 | Sardine | 11.79149 | 17.73109 | 1.801227 | 2.799447 | 0.15 | 0.16 |
| 9 | Sardinelles | 18.0042 | 19.65045 | 2.310539 | 3.112236 | 0.13 | 0.16 |
| 10 | Horse mackerels | 10.00185 | 11.97514 | 3.812705 | 4.733549 | 0.38 | 0.40 |
| 11 | Coastal selacians | 1.208741 | 0.343718 | 0.061412 | 0.076773 | 0.05 | 0.22 |
| 12 | Coastal M | 0.830443 | 0.601779 | 0.121065 | 0.297622 | 0.15 | 0.49 |
| 13 | Coastal S | 4.211359 | 6.68667 |  |  |  |  |
| 14 | Croakers ad | 0.077201 | 0.070635 | 0.003008 | 0.008346 | 0.04 | 0.12 |
| 15 | Croakers juv | 0.003531 | 0.002785 |  |  |  |  |
| 16 | Seabreams ad | 1.69036 | 1.021793 | 0.167036 | 0.281987 | 0.10 | 0.28 |
| 17 | Seabreams juv. | 0.012507 | 0.007336 |  |  |  |  |
| 18 | Catfish ad | 0.600135 | 0.490796 | 0.034008 | 0.148055 | 0.06 | 0.30 |
| 19 | Catfish juv | 0.001666 | 0.001246 |  |  |  |  |
| 20 | Shelf selacians | 0.199917 | 0.17086 | 0.010995 | 0.020673 | 0.06 | 0.12 |
| 21 | Shelf L | 0.360019 | 0.487229 | 0.071004 | 0.181375 | 0.20 | 0.37 |
| 22 | Shelf M | 1.550139 | 1.424855 | 0.146013 | 0.284911 | 0.09 | 0.20 |
| 23 | Groupers ad | 0.107764 | 0.018469 | 0.024492 | 0.01773 | 0.23 | 0.96 |
| 24 | Grouper juv | 0.000367 | 0.000103 |  |  |  |  |
| 25 | Sparids ad | 1.290003 | 1.121018 | 0.014 | 0.049836 | 0.01 | 0.04 |
| 26 | Sparids juv | 0.010753 | 0.009952 |  |  |  |  |
| 27 | Scianids | 0.214149 | 0.033206 | 0.016548 | 0.005362 | 0.08 | 0.16 |
| 28 | Shelf soles | 0.349963 | 0.25992 | 0.008999 | 0.015167 | 0.03 | 0.06 |
| 29 | Shelf S | 6.197166 | 13.73088 | 0.005002 | 0.020918 | 0.00 | 0.00 |
| 30 | Octopus vulgaris | 1.35745 | 0.384884 | 0.874911 | 0.678659 | 0.64 | 1.76 |
| 31 | Cephalopods | 0.985809 | 0.31217 | 0.251381 | 0.151324 | 0.26 | 0.48 |
| 32 | BA L crustaceans | 9.124096 | 5.993826 |  |  |  |  |
| 33 | BA molluscs | 17.86009 | 19.3619 |  |  |  |  |
| 34 | BA worms | 5.319961 | 4.142092 |  |  |  |  |
| 35 | BA crustaceans | 1.140169 | 0.502134 |  |  |  |  |
| 36 | BA other inverts | 0.570191 | 0.661913 |  |  |  |  |
| 37 | BA meiobenthos | 2.08967 | 2.376096 |  |  |  |  |
| 38 | shelf L crustaceans | 8.106485 | 6.285361 | 0.005004 | 0.009989 | 0.000617 | 0.001589 |
| 39 | shelf molluscs | 26.21321 | 20.85251 | 3.01E-06 | 4.52E-06 | 1.15E-07 | 2.17E-07 |
| 40 | shelf worms | 31.77156 | 24.25814 |  |  |  |  |
| 41 | shelf crustaceans | 8.035142 | 3.894845 |  |  |  |  |
| 42 | shelf other inverts | 17.21303 | 15.76241 |  |  |  |  |
| 43 | shelf meiobenthos | 8.909588 | 8.506203 |  |  |  |  |
| 44 | mesozooplankton | 55.07933 | 54.8538 |  |  |  |  |
| 45 | macrozooplankton | 3.410281 | 3.048343 |  |  |  |  |
| 46 | BA mesozooplankton | 1.779772 | 1.793211 |  |  |  |  |
| 47 | BA macrozooplankton | 2.500826 | 2.286842 |  |  |  |  |
| 48 | BA phytoplankton | 5.886206 | 5.880416 |  |  |  |  |
| 49 | phytoplankton | 67.82123 | 68.7425 |  |  |  |  |

## Figure S1. Simplified food web structure showing only the trophic links for which the absolute value of the MTI impact is higher than 0.1.

A=adult, J=juvenile, cat=catfish, cephal=cephalopods, croak=croakers, grou=groupers, hmack= horse mackerel, meag=meagre, sarlle=sardinelle, seab=seabreams,s el=selaciens, spar=sparids. Macrozooplankton includes both shelf and BA groups. Coastal fish in italics, migratory underlined

# References

1. Christensen V, Pauly D (1992) Ecopath II- a software for balancing steady-state ecosystem models and calculating network characteristics. Ecol Model 61: 169-185.

2. Christensen V, Walters CJ (2004) Ecopath with Ecosim: methods, capabilities and limitations. Ecol Model 172: 109-139.

3. Walters CJ, Christensen V, Pauly D (1997) Structuring dynamic models of exploited ecosystems from trophic mass-balance assessments. Rev Fish Biol Fish 7: 139-172.

4. Allen RR (1971) Relation between production and biomass. J Fish Res Bd Canada 28: 1573-1581.

5. Walters C, Kitchell JF (2001) Cultivation/depensation effects on juvenile survival and recruitment: implications for the theory of fishing. Can J Fish Aquat Sci 58: 39-50.

6. Gascuel D, Labrosse P, Meissa B, Ould Taleb Ould Sidi MM, Guénette S (2007) Decline of demersal resources in North-West Africa: an analysis of Mauritanian trawl survey data over the last 25 years. Afr J Mar Sci 29: 331-345.

7. Meissa B, Gascuel D, Rivot E (2013) Assessing stocks in data-poor African fisheries: a case study on the white grouper (*Epinephelus aeneus*) of Mauritania. Afr J Mar Sci 35: 253-267.

8. ould Taleb ould Sidi MM (2005) Les ressources de petits pélagiques en Mauritanie et dans la zone nord ouest africaine: variabilité spatiale et temporelle dynamique et diagnostic [PhD]. Rennes, France: Ecole Nationale Supérieure Agronomique de Rennes. 276 pp.

9. Madureira M (2009) Contribution à l'étude de l'importance du Banc d'Arguin pour la courbine. Available at <http://www.lafiba.org/index.php/fr/documentation/boite_a_outils/articles_scientifiques_posters>. Accessed on 20 June 2011.

10. ould Mohamed Vall M (2004) Etude de la dynamique des systèmes d'exploitations et de l'écobiologie de la reproduction, de trois mugilidés: *Mugil cephalus* (Linnaeus, 1758), *Liza aurata* (Perugia, 1892) et *Mugil capurrii* (Risso, 1810), analyse de leurs strategies d'occupations des secteurs littoraux mauritaniens et de leurs possibilités d'aménagement [PhD]. Nice, France: Univeristé de Nice, Sophia Antipolis. 146 pp.

11. Palomares MLD, Pauly D (1998) Predicting food consumption of fish populations as functions of mortality, food type, morphometrics, temperature and salinity. Mar Freshwater Res 49: 447-453.

12. Christensen V, Pauly D (1992) A guide to the Ecopath software system (version 2.1). ICLARM. 6 72 pp.

13. Pauly D (1980) On the interrelationships between natural mortality, growth parameters, and mean environmental temperature in 175 fish stocks. J Cons Int Explor Mer 39: 175-192.

14. Hoenig (1983) Empirical use of longevity data to estimate mortality rates. Fish Bull 82: 898-903.

15. Labrosse P, Brahim K, Taleib OM, Gascuel D, editors (2010) Evaluation des ressources et aménagement des pêcheries de la ZEE Mauritanienne. Actes du sixième Groupe de travail de l’IMROP. Nouhadibou, Mauritanie: IMROP. 267 p.

16. Brahim K, Jouffre D, Guitton J, Kide SO, ould Ejiwan M. Evolution de la pêche industrielle démersale de 1991 à 2005. In: Labrosse P, Brahim K, Ould Taleb ould Sidi M, Gascuel D, editors; 2010; Nouadhibou, Mauritanie. Rapport du sixième groupe de travail de l'IMROP. pp. 43-56.

17. Morissette L, Kaschner K, Gerber LR (2010) Ecosystem models clarify the trophic role of whales off Northwest Africa. Mar Ecol Prog Ser 404: 289-302.

18. ould Taleb ould Sidi MM, Guénette S (2003) Modèle trophique de la ZEE mauritanienne: comparaison des deux périodes (1987 et 1998). In: Palomares MLD, Vakily JM, Pauly D, editors. Vancouver, BC: UBC Fish Centre Res Rep 12 (7) pp. 12-38.

19. Trites A, Heise K (1996) Marine mammals. In: Pauly D, Christensen V, Haggan N, editors. Mass-Balance Models of North-eastern Pacific ecosystems. Vancouver BC: UBC Fish Centre Res Rep.4 (1) pp. 25-30.

20. Pauly D, Trites AW, Capuli E, Christensen V (1998) Diet composition and trophic levels of marine mammals. ICES J Mar Sci 55: 467-481.

21. Northridge SP (1984) World review of interactions between marine mammals and fisheries. Fisheries and Agriculture Organization. 251 190 pp.

22. Pinela AM, Borrell A, Cardona L, Aguilar A (2010) Stable isotope analysis reveals habitat partitioning among marine mammals off the NW African coast and unique trophic niches for two globally threatened species. Mar Ecol Prog Ser 416: 295-306.

23. Wolff WJ, Smit CJ (1990) The Banc d'Arguin, Mauritania, as an environment for coastal birds. Ardea 78: 17-38.

24. Zwarts L, van der Kamp J, Overdijk O, van Spanje T, Veldkamp R, et al. (1997) Wader count of the Baie d'Arguin, Mauritania, in February 1997. Wader Study Group Bull 86: 70-73.

25. Nilsson SG, Nilsson IN (1976) Number, food, consumption, and fish predation by birds in Lake Mockeln, Southern Sweden. Ornis Scand 7: 61-70.

26. Nelson B (1979) Seabirds;Their biology and ecology. New York: A and W Publishers Inc. 224 pp.

27. Sæther B-E (1989) Survival Rates in Relation to Body Weight in European Birds. Ornis Scand 20: 13-21.

28. Bauchau V, Horn H, Overdijk O (1998) Survival of spoonbills on Wadden Sea islands. J Avian Biol 29: 177-182.

29. Robert M, Faraj A, McAllister MK, Rivot E (2010) Bayesian state-space modelling of the De Lury depletion model: strengths and limitations of the method, and application to the Moroccan octopus fishery. ICES J Mar Sci 67: 1272-1290.

30. Gonçalves JM (1991) The octopoda (Mollusca: Cephalopoda) of the Azores. Arquipel Life Mar Sci 9: 75-81.

31. Hanlon RT, Messenger JB (1996) Cephalopod behaviour. Cambridge: Cambridge University Press. 232 pp.

32. Pierce GJ, Boyle PR, Hastie LC, Key L (1994) The life history of *Loligo forbesi* (Cephalopoda: Loliginidae) in Scottish waters. Fish Res 21: 17-41.

33. Rost Martins H (1982) Biological studies of the exploited stock of *Loligo forbesi* (Mollusca: Cephalopoda) in the Azores. J Mar Biol Assoc UK 62: 799-808.

34. Froerman YM (1984) Feeding spectrum and trophic relationship of short-finned squid (*Illex illecebrosus*) in the Northwest Atlantic. NAFO Sci Coun Studies 7: 67-75.

35. Kuipers BR, Witte HJ, Gonzalez SR (1993) Zooplankton distribution in the coastal upwelling system along the Banc d'Arguin, Mauritania. Hydrobiologia 258: 133-150.

36. Brey T (2002) Population dynamics in benthic invertebrate, a virtual handbook. <http://www.thomas-brey.de/science/virtualhandbook/navlog/index.html>.

37. Aydin KY, MacFarlane GA, King JR, Megery BA (2003) PICES-GLOBEC international program on climate change and carrying capacity; The BASS/MODEL report on trophic models of the subarctic Pacific Basin Ecosystems. Sidney, BC: North Pacific Marine Scienve Organization (PICES). 25 93 pp.

38. Corkett CJ, McLaren IA (1978) The biology of *Pseudocalanus*. Adv Mar Biol 17: 1-454.

39. Guénette S (2005) Model of the Southeast Alaska. In: Guénette S, Christensen V, editors. Foodweb models and data for studying fisheries and environmental impact on Eastern Pacific ecosystems. Vancouver, BC, Canada: Fisheries Centre, University of British Columbia.13 (1) pp. 106-178.

40. Duineveld GCA, de Wilde PAWJ, Berghuis EM, Kok A (1993) The benthic infauna and benthic respiration off the Banc d'Arguin (Mauritania, Northwest Africa). Hydrobiologia 258: 107-117.

41. Wolff WJ, Duiven AG, Esselink P, Gueye A, Meijbom A, et al. (1993) Biomass of macrobenthic tidal flat fauna of the Banc d'Arguin, Mauritania. Hydrobiologia 258: 151-163.

42. Schaffmeister BE, Hiddink JG, Wolff WJ (2006) Habitat use of shrimps in the intertidal and shallow subtidal seagrass beds of the tropical Banc d'Arguin, Mauritania. J Sea Res 55: 230-243.

43. Jarre-Teichmann A, Guénette S (1996) Invertebrate benthos. In: Pauly D, Christensen V, editors. Mass-balance models of North-eastern Pacific ecosystems. Vancouver, BC: UBC Fish Centre Res Rep.4 (1) pp. 38-39.

44. Gascuel D, Morissette L, Palomares MLD, Christensen V (2008) Trophic flow kinetics in marine ecosystems: Toward a theoretical approach to ecosystem functioning. Ecol Model 217: 33-47.

45. Gerlach SA (1971) On the importance of marine meiofauna for benthos communities. Oecologia 6: 176-190.

46. Wolff WJ, Van Der Land J, Nienhuis PH, de Wilde PAWJ (1993) The functionning of the ecosystem of the Banc d'Arguin, Mauritania: a review. Hydrobiologia 258: 211-222.

47. Pauly D, Christensen V (1995) Primary production required to sustain global fisheries. Nature 374: 255-257.

48. Vermaat JE, Beijer JAJ, Gijlstra R, Hootsmans MJM, Philippart CJM, et al. (1993) Leaf dynamics and standing stocks of intertidal *Zostera noltii* Hornem. and *Cymodocea nodosa* (Ucria) Ascherson on the Banc d'Arguin (Mauritania). Hydrobiologia 258: 59-72.

49. Bondavalli C, Ulanowicz RE (1999) Unexpected Effects of Predators Upon Their Prey: The Case of the American Alligator. Ecosystems 2: 49-63.

50. Trites A, Pauly D (1998) Estimating mean body masses of marine mammals from maximum body lengths. Can J Zool 76: 886-896.

51. Leopold MF (1993) Seabirds in the shelf edge waters bordering the Banc d'Arguin, Mauritania. Hydrobiologia 258: 197-210.

52. Campredon P (2000) Between the Sahara and the Atlantic, Banc d'Arguin National Park, Mauritania. la Tour du Valat, Arles, France: FIBA. 124 pp.

53. Granadeiro JP (1991) The breeding biology of Cory's shearwater *Calonectris diomedea borealis* on Berlenga Island, Portugal. Seabird 13: 30-39.

54. Russell RW. Comparative demography and life history tactics of seabirds: Implications for conservation and marine monitoring. In: Musick JA, editor; 1999; Monterey, California. Am. Fish. Soc. Symp. pp. 51-76.

55. Dick WJA, Pienkowski MW (1979) Autumn and early body weights of waders in the north-west Africa. Ornis Scand 10: 117-123.

56. Sæther B-E (1987) The Influence of body weight on the covariation between reproductive traits in European birds. Oikos 48: 79-88.

57. Hedenström A (1998) Flight speed of Ross's gull *Rhododtethia rosea* and Sabine's gull *Larus sabini*. Arctic 51: 283-285.

58. Brey T (2009) The Virtual Handbook Alfred Wegener Institute: Available at <http://www.thomas-brey.de/science/virtualhandbook/navlog/index.html>. Accessed on June 2010.

59. Tita G, Vincx M, Desrosiers G (1999) Size, spectra, body width and morphotypes os intertidal nematodes: an ecological interpretation. J Mar Biol Assoc UK.

60. Danovaro R, Dell'Anno A, Martorano D, Parodi P, Marrale ND, et al. (1999) Seasonal variation in the biochemical composition of deep-sea nematodes: bioenergetic and methodological considerations. Mar Ecol Prog Ser 179: 273-283.

61. Herman PMJ, Heip C (1983) The respiration of five brackish-water harpacticoid copepod species. J Exp Mar Biol Ecol 71: 249-256.
